# Novel chemokine related LncRNA signature correlates with the prognosis, immune landscape, and therapeutic sensitivity of esophageal squamous cell cancer

**DOI:** 10.1186/s12876-023-02688-5

**Published:** 2023-04-20

**Authors:** Zhe Zhang, Jian Wang, Wei Han, Li Zhao

**Affiliations:** 1 Department of Gastroenterology, Tangdu Hospital, Fourth Military Medical University, No. 1 Xinsi Road, Xi’an, China; 2 Department of Neurosurgery, Tangdu Hospital, Fourth Military Medical University, Xi’an, China

**Keywords:** Esophageal squamous cell cancer, ESCC, Chemokine related lncRNA, Prognosis

## Abstract

**Background:**

Esophageal squamous cell carcinoma (ESCC) is closely correlated with malignant biological characteristics and poor survival. Recently, chemokines have been reported to be involved in the progression of tumors, and they can also regulate the tumor microenvironment. However, it is unclear whether chemokine-related long noncoding RNAs (lncRNAs) affect the prognosis of ESCC.

**Methods:**

We downloaded RNA-seq and clinical data from the Gene Expression Omnibus (GEO database. Chemokine-related lncRNAs were screened by differential analysis and Pearson correlation analysis. Then, prognosis-related lncRNAs were screened by using univariate COX regression, and risk models were constructed after the least absolute shrinkage and selection operator (LASSO) regression and multivariate COX regression. The predictive value of the signature was assessed using Kaplan–Meier test, time-dependent receiver operating characteristic (ROC) curves, decision curve analysis (DCA) and calibration curve. Moreover, a nomogram to predict patients’ 1-year 3-year and 5-year prognosis was constructed. Gene set enrichment analyses (GSEA), Gene Ontology/Kyoto Encyclopedia of Genes and Genomes (GO/KEGG), evaluation of immune cell infiltration, and estimation of drug sensitivity were also conducted.

**Results:**

In this study, 677 chemokine-related lncRNAs were first obtained by differential analysis and Pearson correlation. Then, six chemokine-related lncRNAs were obtained by using univariate COX, LASSO and multivariate COX to construct a novel chemokine-related lncRNAs risk model. The signature manifested favorable predictive validity and accuracy both in the testing and training cohorts. The chemokine-related signature could classify ESCC patients into two risk groups well, which indicated that high-risk group exhibited poor prognostic outcome. In addition, this risk model played an important role in predicting signaling pathways, immune cell infiltration, stromal score, and drug sensitivity in ESCC patients.

**Conclusions:**

These findings elucidated the critical role of novel prognostic chemokine-related lncRNAs in prognosis, immune landscape, and drug therapy, thus throwing light on prognostic evaluation and therapeutic targets for ESCC patients.

**Supplementary Information:**

The online version contains supplementary material available at 10.1186/s12876-023-02688-5.

## Introduction

As the seventh most common type of tumor [1], esophageal cancer (EC) is histologically classified into two subtypes: esophageal squamous cell carcinoma (ESCC) and esophageal adenocarcinoma (EAC) [2]. To be specific, ESCC accounts for > 90% of EC, thus it becomes the main EC histologic type, especially in high-incidence areas of Asia [3]. The past decades have witnessed the major progress in diagnosis and management of ESCC, such as, surgical techniques, radiotherapy, and chemotherapy. However, most ESCC patients still have suffered poor prognosis mainly caused by delay in diagnosis [4, 5]. Consequently, it is urgent to identify reliable biomarkers associated with the prognosis of ESCC in an aim to promote disease stratification and therapeutic measure.

Many noncoding RNAs including long noncoding RNAs (lncRNAs) could also be identified as diagnostic biomarkers or prognostic factors in ESCC [6, 7]. LncRNAs are composed of sequences > 200 bp, and lack protein-coding capabilities. Numerous lncRNAs play a crucial role in the tumor development, such as participating in gene regulation and various biological functions at the transcriptional, post-transcriptional and epigenetic levels [8–10]. In addition to gene regulation, lncRNAs are also involved in the regulation of many biological processes related to tumorigenesis [11]. Mounting evidence suggested that lncRNAs play an important role in prognosis prediction in ESCC. For example, prognostic signatures such as lactic acid metabolism, ferroptosis-related lncRNA, and m7G-related lncRNA were proved to have favorable prognosis prediction in ESCC [12–14]. In addition, lncRNA based model can be used as therapeutic target, which could forecast the effect of immunotherapy or chemotherapy [15, 16].

Chemokines are a large class of cytokines with chemotactic activity. Chemokines have been reported to modulate cancer progression and may serve as therapeutic targets [17]. For example, CC and CXC chemokines can promote tumor angiogenesis, which further promotes tumor growth and metastasis [17]. Previous study also indicated that chemokine-related genes may exert important effects in the infiltration of various immune cells and the tumor microenvironment (TME), thereby affecting tumor progression. Specifically, they can induce anti-tumor immune responses by increasing interferon-γ expression by regulating T cell infiltration, and generate a tumor-promoting microenvironment by recruiting regulatory T cells (Treg) or tumor-associated macrophages (TAMs) [18, 19]. For example, CCL24 can contribute to the progression of multiple cancers through M2 macrophage polarization, angiogenesis, invasion and migration, and eosinophil recruitment [20].

Indeed, the identification of the TME is associated with tumorigenesis, progression, and novel immunotherapeutic targets. TME may also provide meaningful clues for future treatment of ESCC, particularly immunotherapy. At present, Clinical and biological function of chemokine-related lncRNA still need to be further investigated. Associations between chemokine-related lncRNAs and the immune microenvironment of ESCC have yet not been reported. Therefore, in this study, we aimed to construct and validate a lncRNA signature model consisting of six chemokine-related lncRNAs and other clinical indicators derived from the Gene Expression Omnibus (GEO) database. Apart from that, we also planned to clarify the correlation between chemokine-related signature and immune cell infiltration, and further explore potential chemotherapeutic agents.

## Methods

### Data collection and pre-processing

The GSE53624 and GSE53622 datasets were obtained from the GEO database (https://www.ncbi.nlm.nih.gov/geo/). In brief, the GSE53624 dataset includes 119 ESCC patients and 119 paired adjacent normal samples while GSE53622 dataset incorporates 60 ESCC patients and 60 paired adjacent normal samples. In each dataset, patients lacking complete follow-up information and without survival days were excluded. All samples were divided into training and validation sets in a ratio of 75 to 25%. The aforementioned datasets were generated with Agilent-038314 (GPL18109). We gained the expression values of lncRNAs from ESCC cohorts through re-annotating microarray probes [21–23]. We also used the “sva” R package to batch and normalize lncRNA expression profiles. Then, expression levels of lncRNAs in tumor and paired adjacent normal groups were analyzed by the limma package, respectively, with the same parameters (|logFC|> 1, FDR < 0.05), and 736 DElncRNAs were identified. 64 chemokine genes, defined as chemokines or chemokine receptors, were gathered from previous literature [24–27] (Table S1). Based on these 64 chemokines, we screened chemokine-related DElncRNAs by intersecting them with differentially expressed lncRNAs (DElncRNAs). By co-expression analysis, the threshold was set to the correlation coefficient > 0.3 and *P*-value < 0.001, and 677 chemokine-related DElncRNAs were identified. Subsequently, univariate analysis was performed to determine prognosis-related chemokine-related DElncRNAs. A total of 39 prognostic chemokine-related DElncRNAs were identified.

### Establishment and validation of the risk model

In brief, the training set was used to construct the risk model while the validation set was used for the validation of the risk model. Firstly, the least absolute shrinkage and selection operator machine learning algorithm (LASSO) regression analysis [28] and multivariate cox regression were utilized to construct the lncRNA risk model. Finally, 6 chemokine-related lncRNAs were used to establish the risk model. The calculation formula of the risk score can be seen as follows:

$$\mathrm{Risk\ score }\ \left(\mathrm{patients}\right) = \sum_{i=1}^{n}\mathrm{Coefi }*\mathrm{ Expi}$$

In this formula, Coefi represents the coefficient whereas Expi represents the expression value of chemokine-related lncRNA, respectively. The median value of risk score was used as a cut-off value to separate the samples into high or low expression groups. Kaplan–Meier survival analysis was used to determine the over survival (OS) difference between these two groups. A time-dependent receiver operating characteristic (ROC) curve was plotted to detect the predictive ability of the risk model. Then, heatmap was used to visualize the expression of the chemokine-related lncRNAs in the model. The predictive power of risk scores in age, gender, and TNM stage sub-groups was validated by stratified survival analysis. All analyses were further performed in the validation set. R package of “survivalROC”,“survival”, “survminer” and “pheatmap” were used in the validation of the risk model.

### Prognostic value of the risk model

Univariate analysis and multivariate analysis were used to detect the independent prognostic value of the risk model. Kaplan–Meier survival analysis was used to determine the over survival (OS) difference among patients with different clinical characteristics. The ROC and calibration curve were performed to validate the predictive ability of the risk model. To facilitate the prediction of 1-, 3-, and 5- year overall survival (OS) probability in ESCC patients, a nomogram was then developed using the “survival” and “regplot” R packages. The calibration curve was acquired to assess the accuracy of the nomogram by using “rms” package of R.

### Functional enrichment analysis

Gene set enrichment analyses (GSEA) were performed to define the lncRNAs signatures in the Kyoto Encyclopedia of Genes and Genomes (KEGG) [29–31]. Subsequently, we obtained and evaluated the difference in immune-related pathways between the high-risk group and low-risk group through the single-sample gene set enrichment analysis (ssGSEA) [32]. In ssGSEA analysis, the R packages of “limma”, “GSVA”, “GSEABase”, “ggpubr”, “reshape2” were used.

### Evaluation of immune cell infiltration

To predict the proportion of infiltrating immune cells in tumors, we used the CIBERSORT bioinformatic computational tool [33]. The reliability of the deconvolution method was used for transcriptional enrichment of immune cell types. and the algorithm used a default signature matrix with perm = 1000 times for analysis. The “corrplot” package was used to visualize the correlation among 22 types of tumor-infiltrating immune cells. Then, we evaluated the correlation between chemokine-related lncRNA and stromal score, immune score, estimate score and tumor purity using the ESTIMATE algorithm. The analysis was visualized by R packages of “ggpubr”, “ggplot2” and “data.table”.

### Estimation of drug response in clinical samples

Drug sensitivity was obtained use the R package “pRRophetic”, which predicted 50% inhibitory concentration (IC50) of common drugs for ESCC. The predictive model was trained on expression profiles and drug response data of solid cancer cell lines by default tenfold cross-validation. Following that, we determined drug sensitivities in different risk groups, and screened for potential therapeutic agents that might affect patient survival.

### Statistical analysis

For continuous variables, the t-test or Wilcoxon test was used to compare the difference between two groups, and one-way ANOVA or Kruskal − Wallis was used to compare the.difference among more than two groups. For categorical variables, χ^2^ test was used to examine the differences between groups. Survival analysis was performed based on Kaplan–Meier and log-rank tests. Statistical significance was considered to be at two-sided *P* < 0.05. All analyses were performed with R version 4.0.2 (http://www.R-project.org).

## Results

**Fig. 1:**
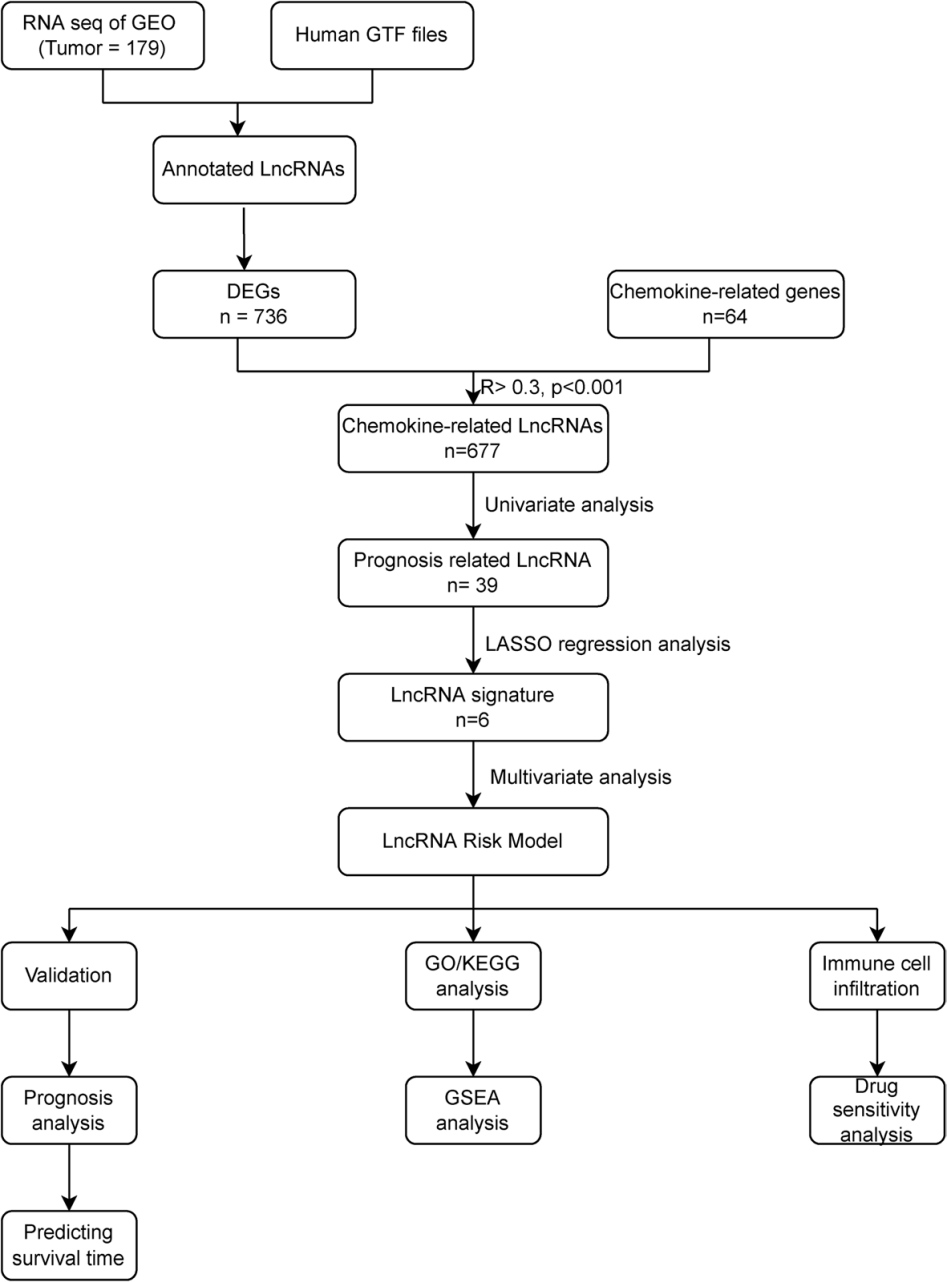
Flow chart of the study. GEO, Gene Expression Omnibus; DEG, differentially expressed gene; LASSO, least absolute shrinkage and selection operator; GO/KEGG, Gene Ontology/Kyoto Encyclopedia of Genes and Genomes; GSEA, Gene set enrichment analyses

**Fig. 2:**
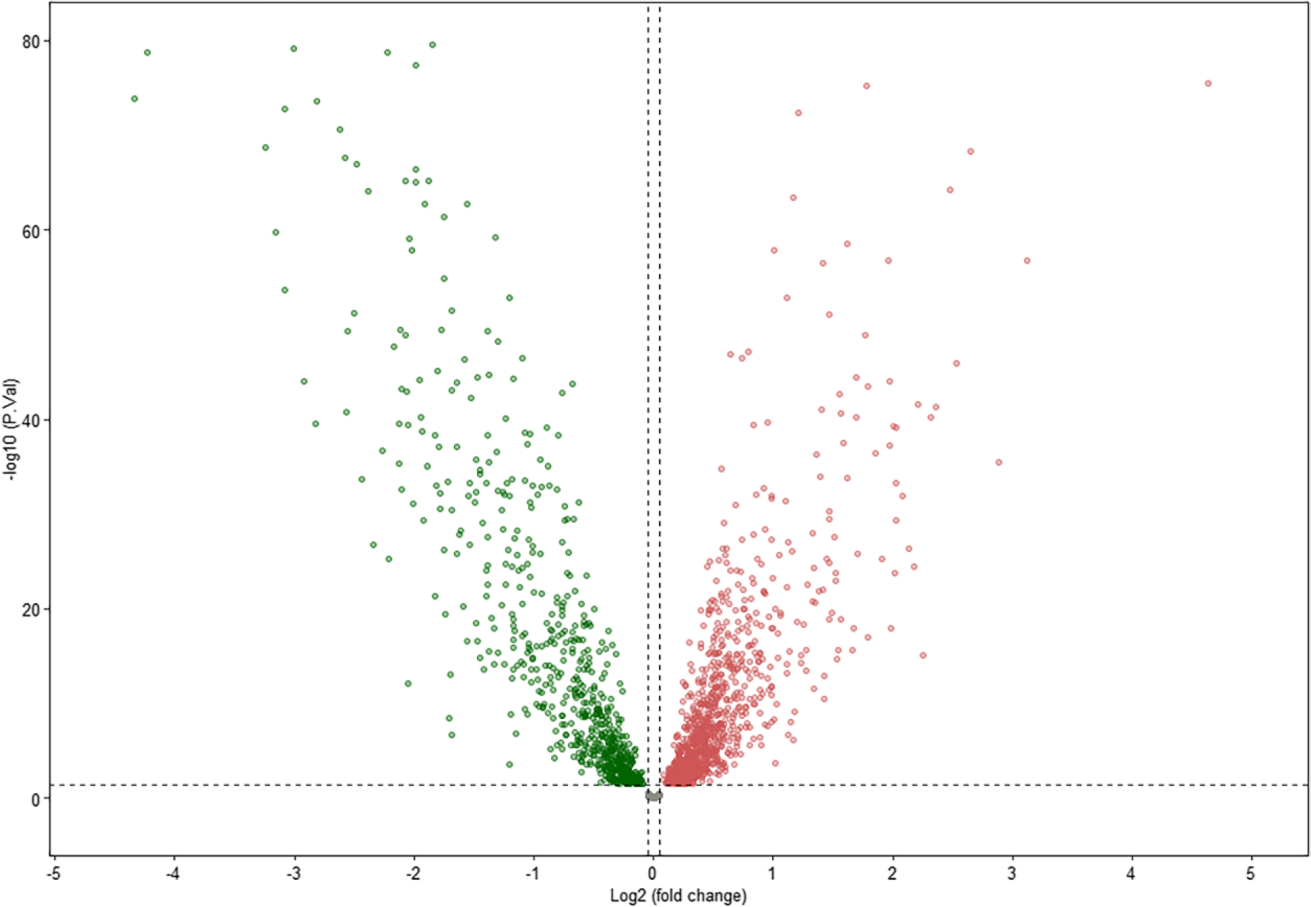
Volcano plot for differential expressed gene analysis

The process of data extraction, processing is shown in Fig. 1. First, we acquired the expression profiling data of 179 tumor samples and corresponding clinical information from GEO. The human GFT file was utilized to annotate the gene symbols in order to acquire the expression data of lncRNAs. We obtained 736 DEGs between tumor and paired adjacent normal groups, of which 375 genes were upregulated and 361 genes were downregulated (Fig. 2). Subsequently, we obtained 64 chemokine-related genes, including chemokines and chemokine receptors from previous literature. After Pearson’s correlation analysis (Pearson ratio > 0.3 and *P* < 0.001), a total of 677 chemokine-related DElncRNAs were identified, and will be used for the subsequent analyses.

### Construction and validation of the risk model

**Fig. 3:**
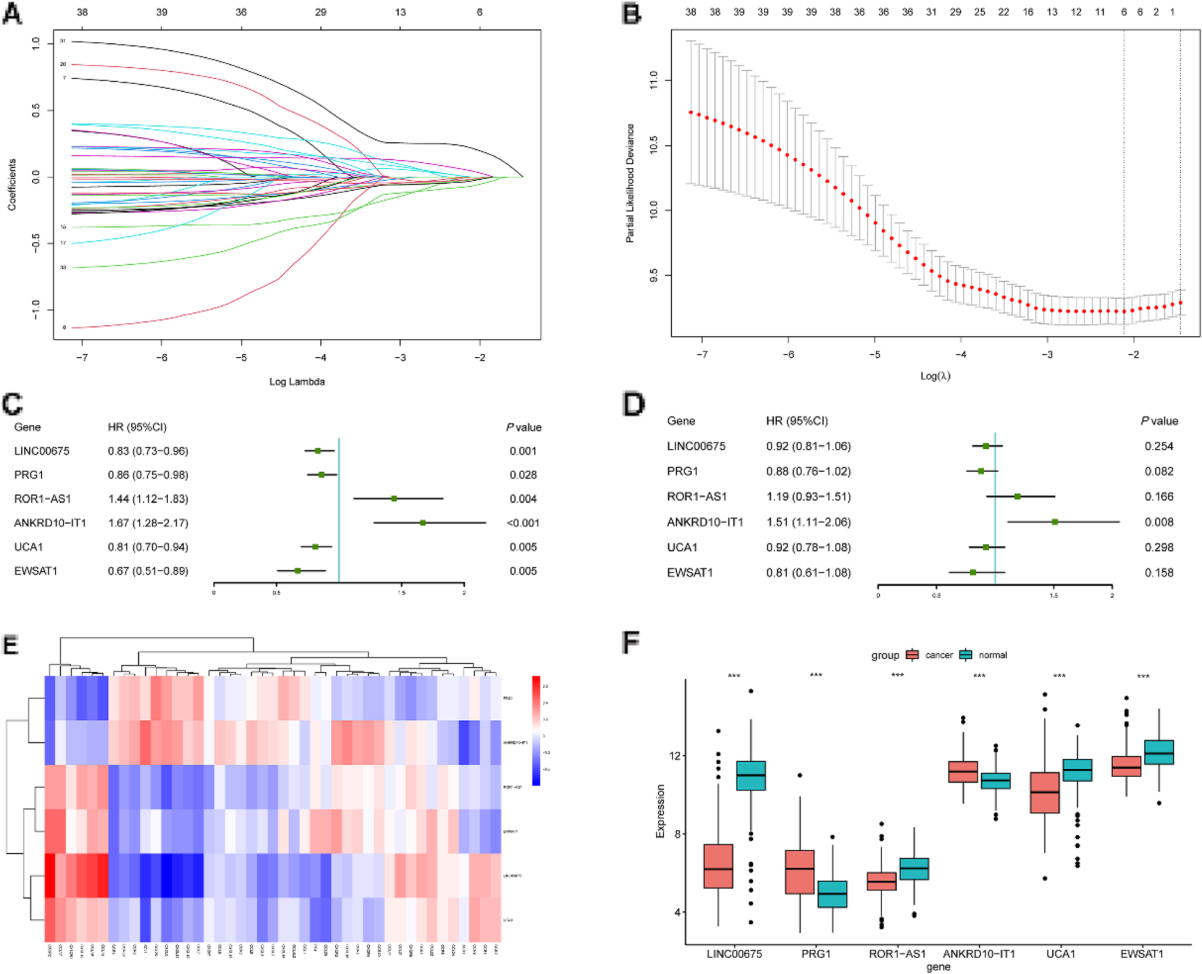
Construction of the risk model. **A**, **B** The process and result of LASSO regression. **C**, **D** Univariate and multivariate analyses were performed to investigate the relationship between six chemokine-related lncRNA and OS. **E** The correlation between chemokines and lncRNAs in the risk model. **F** The expression of six lncRNA in the cancer group and normal group (*p < 0.05, **p < 0.01, ***p < 0.001)

**Fig. 4:**
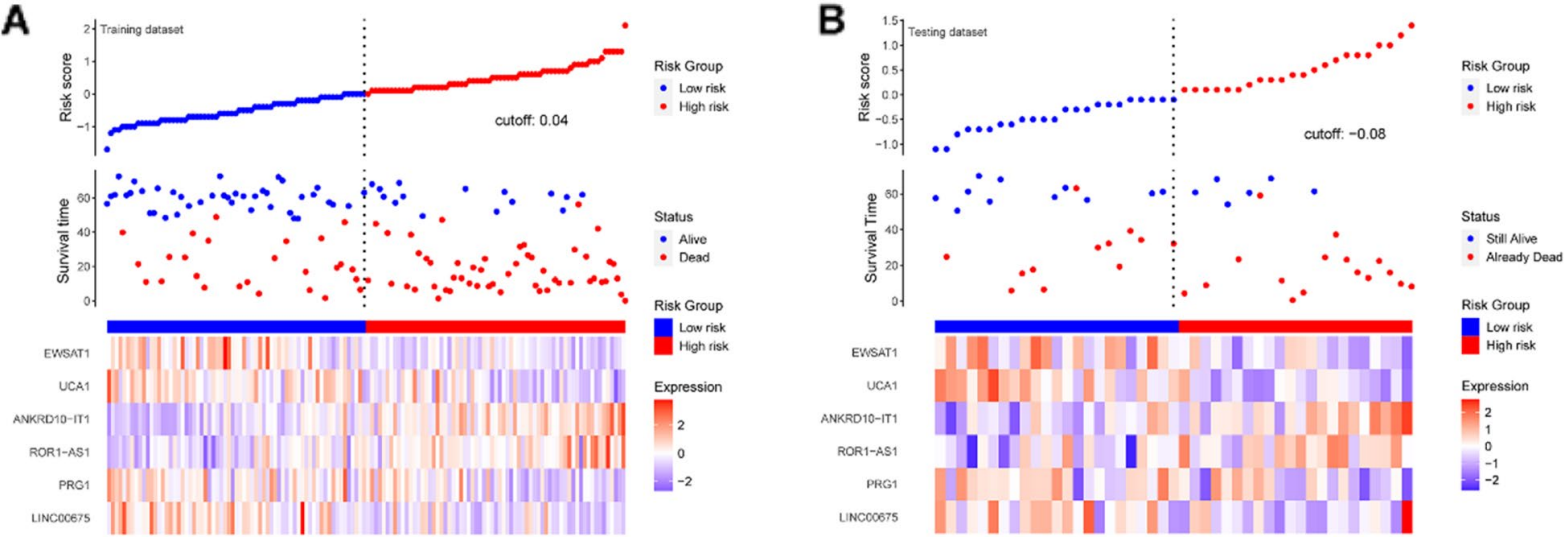
The survival status of the patients in high-risk and low-risk groups. **A** The survival status of the patients in high-risk and low-risk groups in the training dataset. **B** The survival status of the patients in high-risk and low-risk groups in the testing dataset

**Fig. 5:**
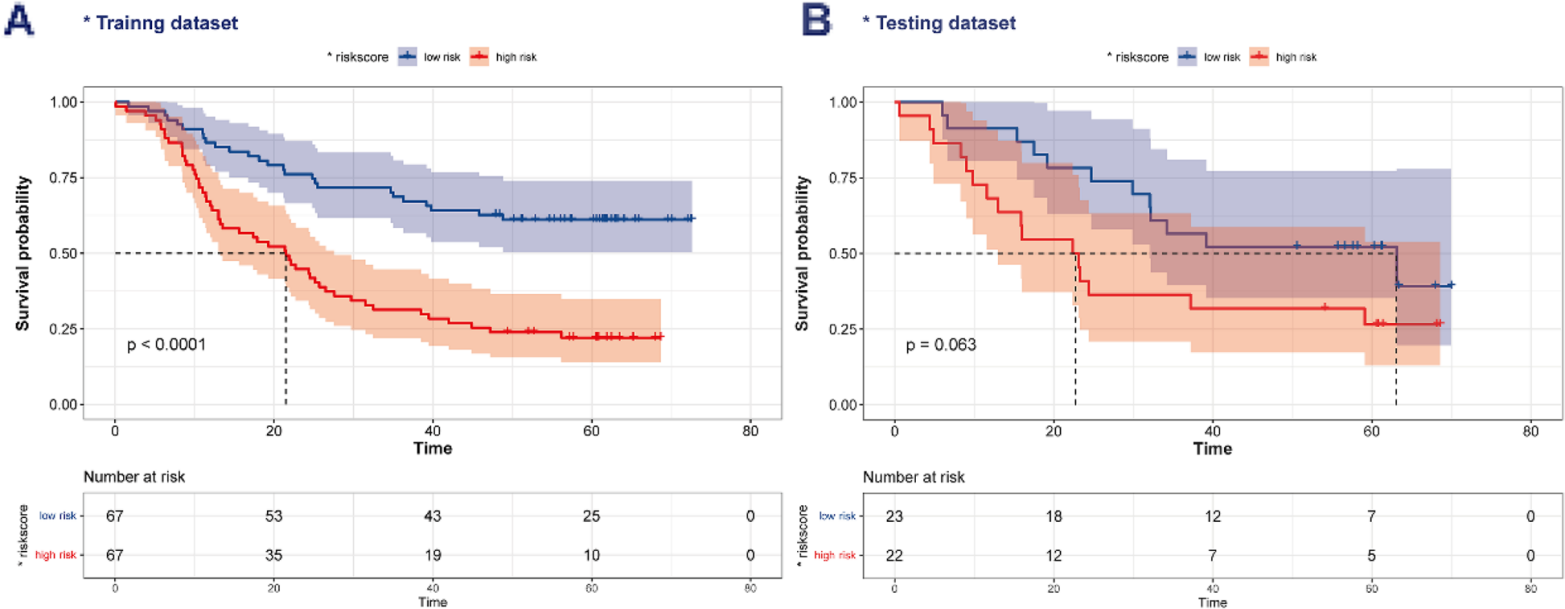
Survival analysis. **A** Survival analysis in the training set. **B** Survival analysis in the testing set

**Fig. 6:**
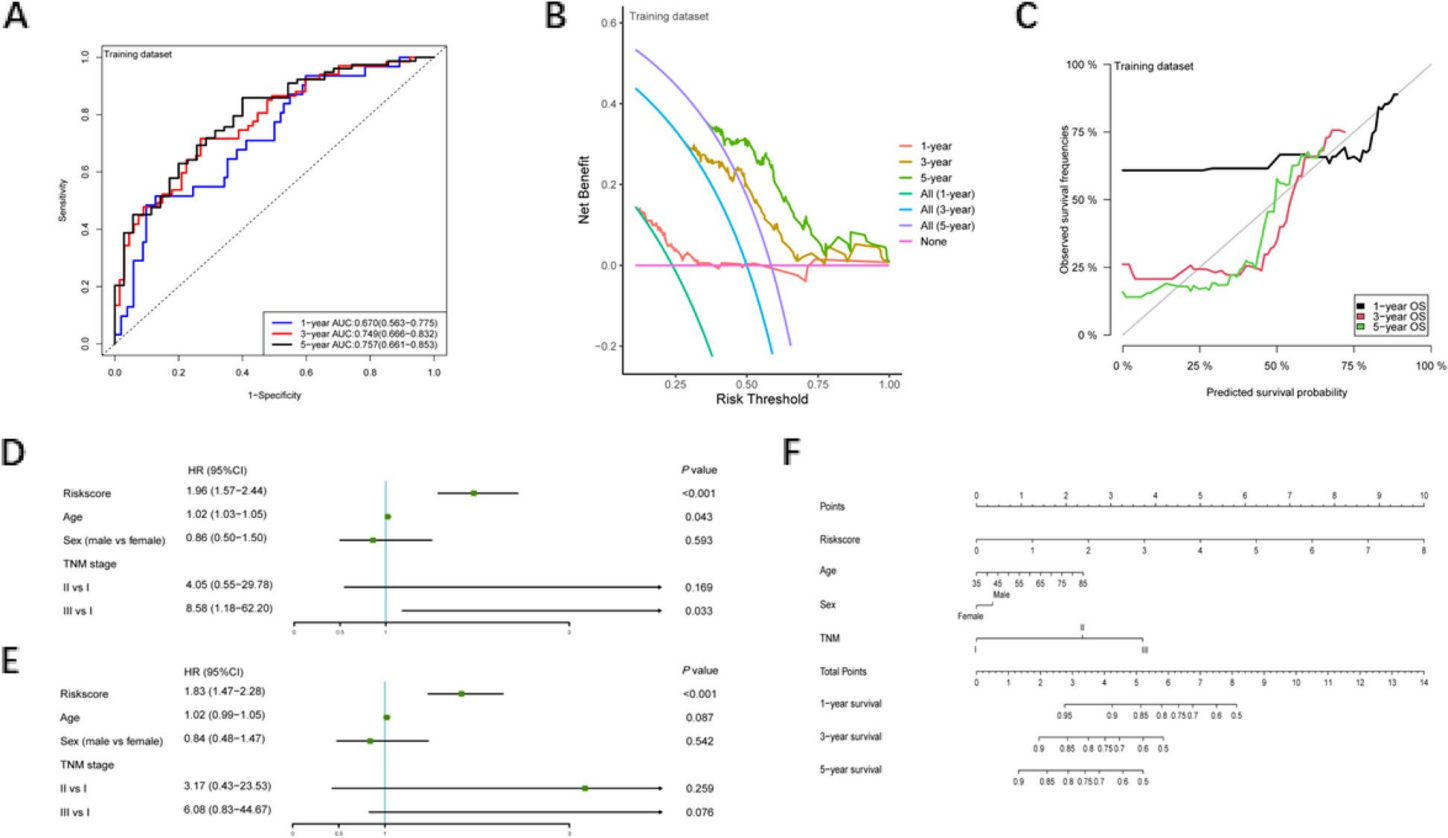
Performance of risk model. **A** ROC of risk model in the training set. **B** DCA of risk model in the training set. **C** Calibration curve in the training set. **D** ROC curves were performed to validate the superiority of the risk score in predicting patient’ survival (Univariate analysis). **E** ROC curves were performed to validate the superiority of the risk score in predicting patient’ survival (Multivariate analysis). **F** Nomogram was plotted for the prediction of overall survival time

After obtaining chemokine-related lncRNAs, we performed univariate cox analysis in order to combine the survival status with lncRNA expression data. 39 prognostic chemokine-related lncRNAs were obtained (Table 1). Then, the training set was used for the establishment of the risk model. First, LASSO regression analysis iterates 500 times to reduce the dimension of data features, which generated 6 optimal candidates (Fig. 3A, B). Forest plot showed the relationship between the six selected lncRNAs and prognosis (Fig. 3C, D). Then, multivariate cox analysis was performed to construct a risk model. By using a heatmap, we visualized the correlation between chemokines and 6 lncRNAs (Fig. 3E). The expression of lncRNAs in the paired adjacent normal and tumor groups is shown in Fig. 3F. According to the median risk score, patients were divided into a high-risk group and a low-risk group (Fig. 4A). The expression of lncRNAs in the low-risk and high-risk group is shown in Fig. S1. To further validate the efficiency of the risk model in predicting the survival of ESCC patients, survival analysis was conducted. And we found that low-risk group patients had a better survival outcome than high-risk patients (Fig. 5A). Next, we tested the predictive ability of the risk model by using a time-dependent receiver operating characteristic (ROC) curve, decision curve analysis (DCA) and calibration curve. For example, the AUC values in the training set were 0.670, 0.749 and 0.757 at one- year, three- year and five- year (Fig. 6A), respectively. In terms of discrimination and calibration, these results revealed that the risk model has enough efficiency in predicting the survival of ESCC patients (Fig. 6B, C). Moreover, we observed that there were more deaths in the high-risk group than in the low-risk group.

**Table 1:** Univariate analysis was performed to evaluate to predict chemokine-related lncRNA and OS

Symbol	HR	95% CI	*P* Value
ACTA2-AS1	1.25	(1.00, 1.55)	0.046
ADAMTS9-AS1	1.28	(1.07, 1.53)	0.006
ANKRD10-IT1	1.56	(1.23, 1.99)	< 0.001
C8orf49	0.72	(0.57, 0.91)	0.007
CYP1B1-AS1	1.22	(1.03, 1.45)	0.02
DLEU7-AS1	1.21	(1.00, 1.45)	0.047
DLX6-AS1	0.74	(0.56, 0.96)	0.025
EWSAT1	0.69	(0.54, 0.88)	0.002
FENDRR	1.19	(1.03, 1.37)	0.02
FLJ40288	0.79	(0.63, 0.99)	0.036
FLJ42351	0.71	(0.54, 0.94)	0.016
HAND2-AS1	1.17	(1.05, 1.29)	0.003
HCG27	0.84	(0.72, 0.98)	0.022
HPYR1	0.79	(0.62, 0.99)	0.042
JMJD1C-AS1	1.21	(1.02, 1.44)	0.028
KCNQ1OT1	1.37	(1.06, 1.76)	0.016
LINC00515	1.23	(1.02, 1.47)	0.03
LINC00623	1.34	(1.09, 1.66)	0.006
LINC00675	0.85	(0.75, 0.95)	0.005
LINC00864	0.72	(0.53, 0.96)	0.028
LINC01266	1.18	(1.02, 1.36)	0.028
LINC01659	0.85	(0.75, 0.96)	0.007
LINC01749	0.75	(0.61, 0.91)	0.004
LINC01829	1.23	(1.03, 1.47)	0.02
LOC100128573	0.86	(0.74, 1.00)	0.048
LOC100505817	0.91	(0.86, 0.97)	0.005
LOC100507373	0.67	(0.48, 0.92)	0.013
MAMDC2-AS1	1.31	(1.05, 1.65)	0.018
MCPH1-AS1	0.75	(0.58, 0.96)	0.021
NDUFB2-AS1	0.68	(0.51, 0.92)	0.011
NUP50-AS1	0.83	(0.71, 0.98)	0.026
PRG1	0.81	(0.72, 0.91)	< 0.001
ROR1-AS1	1.48	(1.20, 1.84)	< 0.001
TINCR	0.78	(0.65, 0.93)	0.006
UCA1	0.83	(0.74, 0.95)	0.005
UG0898H09	1.18	(1.01, 1.38)	0.034
USP12-AS2	0.58	(0.37, 0.91)	0.017
WFDC21P	0.88	(0.79, 0.99)	0.033
WT1-AS	0.47	(0.31, 0.72)	0.001

To further validate the performance of the risk model, we conducted comprehensive analysis in the validation set. First, we also further explored the median value of the risk score in the validation set and found that it could better distinguish between high-risk and low-risk groups (Fig. 4B). Second, we observed that in the validation set, as the risk scores increased, the number of patients at risk decreased at a higher rate (Fig. 5B). Although the *P*-value was not significant, this may be because the validation set had a small sample size of only 45. We also did subgroup analysis in different groups stratified by age, gender, and TNM stage and found that the most significant difference in survival between the high-risk and low-risk groups was observed in the female population (Fig. S2). To further explore whether risk model could better predict survival or death, we tested the discrimination and calibration ability of risk model in validation set by using a time-dependent ROC curve, DCA curve and calibration (Figs. S3, S4, S5). As expected, we observed that our risk model had a relatively preferable performance. For example, the calibration curves for the 3- and 5-year survival rates were close to the diagonal line, indicating that the model had good calibration.

### Prognostic value of the risk model

As shown in Table 2, no difference was observed in clinical characteristics between high-risk and low-risk patients, indicating that the baseline conditions of two groups were balanced and comparable. To validate the independent prognostic value of the risk model, univariate analysis and multivariate analysis were performed. We found that the risk score could be used as an independent prognostic variable (Fig. 6D, E). Combined with other clinical characteristics including age, sex and TNM stage, we constructed a predictive model to predict 1-, 3-, 5- year survival in the entire set. ROC, DCA, and calibration curve were also conducted to evaluate the performance of the clinical model both in the training set (Figs. S6, S7, S8) and testing set (Figs. S9, S10, S11). In addition, nomogram was plotted to help physicians to estimate the probability of survival of patients (Fig. 6F).

**Table 2:** Clinical characteristics of the ESCC patients from GEO database

Variable	Low-risk group (*N* = 90)	High-risk group (*N* = 89)	*P* value
Age (years)			0.474
≤ 65	68 (75.56)	62 (69.66)	
> 65	22 (24.44)	27 (30.34)	
Sex			1.000
Female	17 (18.89)	16 (17.98)	
Male	73 (81.11)	73 (82.02)	
Tobacco use			0.799
Yes	56 (62.22)	58 (65.17)	
No	34 (37.78)	31 (34.83)	
Alcohol use			0.503
Yes	56 (62.22)	50 (56.18)	
No	34 (37.78)	39 (43.82)	
Tumor grade			0.027
Moderately	57 (63.33)	41 (46.07)	
Poorly	17 (18.89)	32 (35.96)	
Well	16 (17.78)	16 (17.98)	
T stage			0.098
T1	6 (6.67)	6 (6.74)	
T2	16 (17.78)	11 (12.36)	
T3	59 (65.56)	51 (57.30)	
T4	9 (10.00)	21 (23.60)	
N stage			0.796
N0	44 (48.89)	39 (43.82)	
N1	31 (34.44)	31 (34.83)	
N2	9 (10.00)	13 (14.61)	
N3	6 (6.67)	6 (6.74)	
TNM stage			0.585
1	6 (6.67)	4 (4.49)	
2	41 (45.56)	36 (40.45)	
3	43 (47.78)	49 (55.06)	
Survival status			< 0.001
Alive	52 (57.78)	21 (23.60)	
Dead	38 (42.22)	68 (76.40)	

### Functional enrichment analysis

**Fig. 7:**
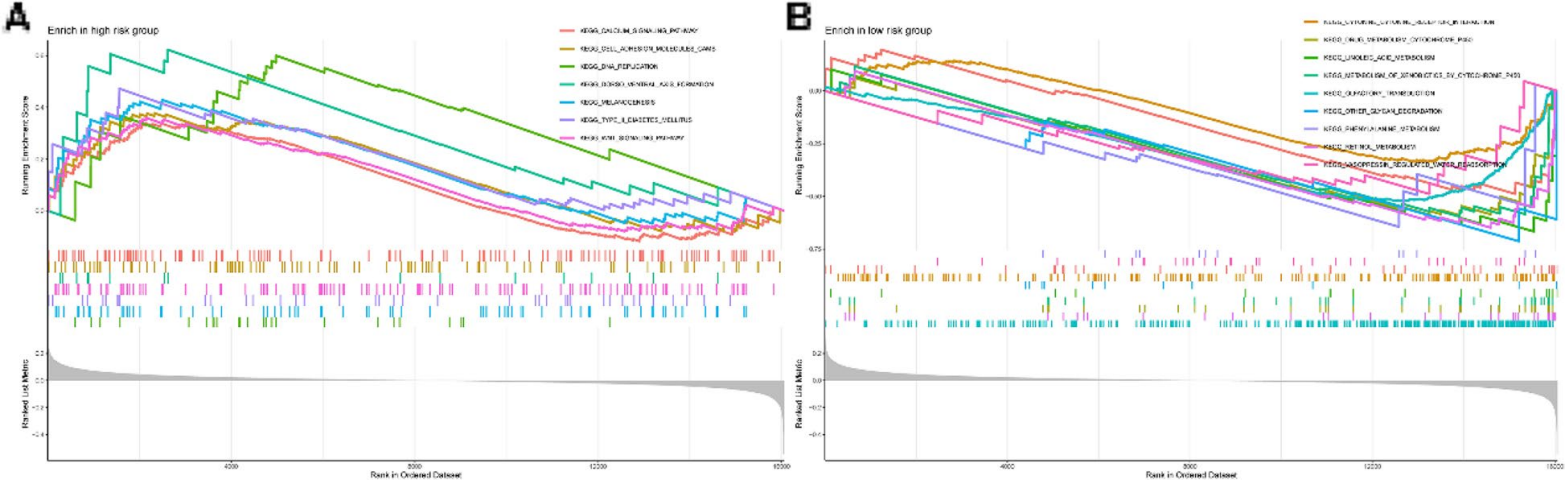
Gene set enrichment analysis based on the chemokine-related lncRNAs risk model. **A** GSEA in the high-risk group. **B** GSEA in the low-risk group

To detect the difference in KEGG enrichment between the low-risk and high-risk patients, gene set enrichment analyses (GSEAs) was performed. Pathways obviously enriched in the high-risk group were calcium signaling, cell adhesion, DNA replication, dorso ventral axis formation, melanogenesis, type II diabetes, and WNT signaling, most of which play crucial roles in tumor development and migration (Fig. 7A, B). To further explore the enriched pathways of DEGs between high-risk and low-risk groups, we performed KEGG enrichment analysis. In total, 40 enriched Gene Ontology GO terms were obtained and the top 20 were shown in Fig. S12, including herpes simplex virus 1 infection, vascular smooth muscle contraction, cytokine-cytokine receptor interaction, signaling pathways regulating pluripotency of stem cell, calcium signaling pathway, WNT signaling pathway and so forth.

### Evaluation of immune cell infiltration

**Fig. 8:**
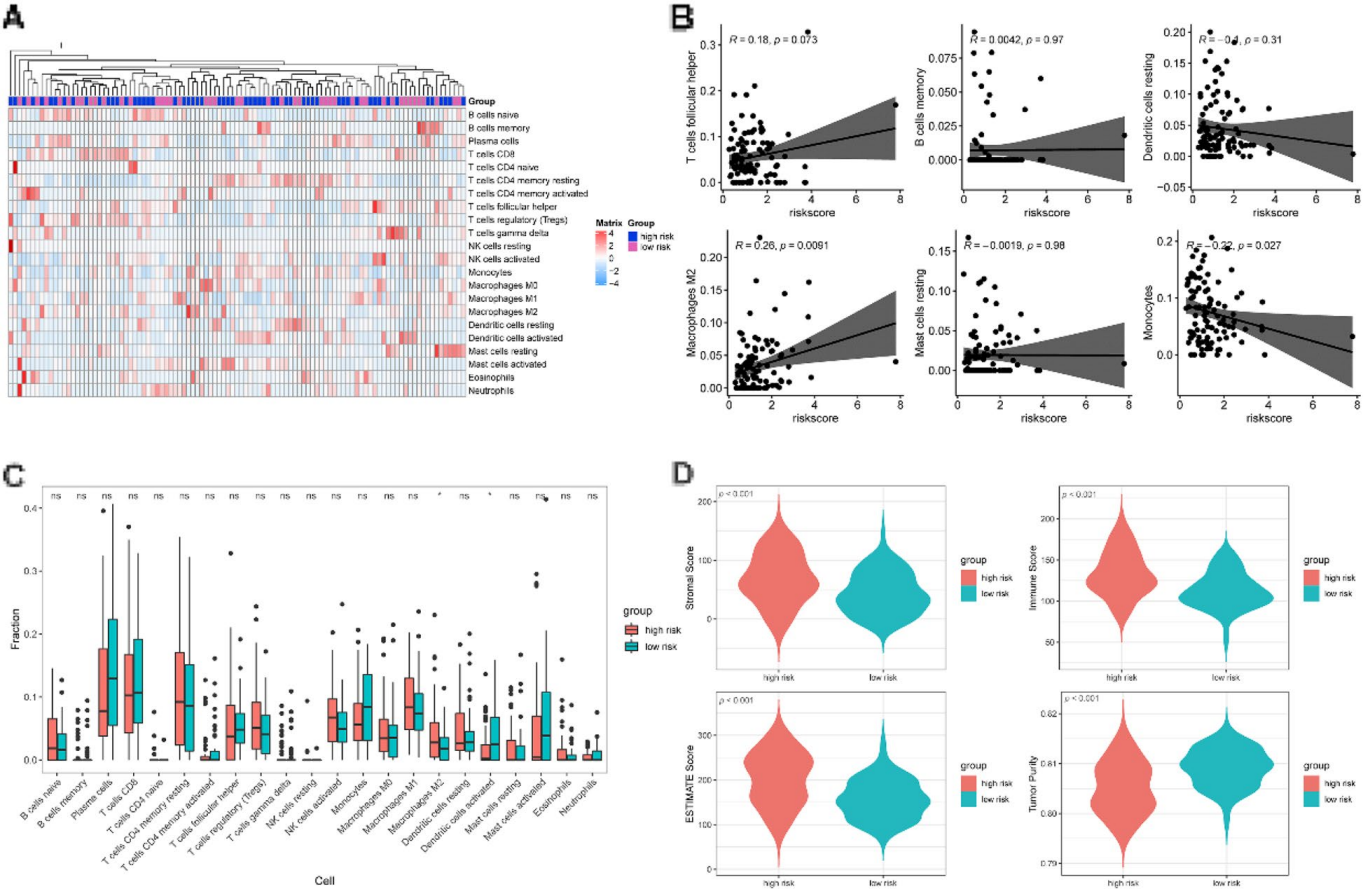
Tumor in filtrating immune cells in ESCC. **A** Correlation between 22 tumors in filtrating immune cells was visualized. **B** Correlation between the risk score and in filtrating level of T cells follicular helper, B cells memory, dendritic cells resting, macrophages M2, mast cells resting and monocytes. **C** Boxplots of differences in immune cell infiltration between high-risk and low-risk groups. **D** Difference in Stromal Score, Immune Score, ESTIMATE Score, and Tumor Purity between high-risk and low-risk groups

Based on the above results, we hypothesized that the low-risk and high-risk groups have different immune microenvironment statuses. Thus, we used the CIBERSORT analysis to calculate the infiltration status. As demonstrated in the Fig. 8A, the infiltration pattern of 22 immune cells differed between low-risk group and high- risk group. Further, we found that risk score had a correlation with macrophages M2 and monocytes (Fig. 8B). In addition, we observed that high-risk group patients had a higher infiltration of macrophages M2 yet a lower infiltration of dendritic cells activated (Fig. 8C). These results suggested an immune-suppressive TME with predicted malignant biological behaviors of ESCC cells in high-risk group. Further, in the TME, the average Immune Score, Stromal Score, and ESTIMATES Score were markedly higher in the high-risk group while the Tumor Purity was markedly higher in the low-risk group, which predicted a better prognosis. (Fig. 8D).

### Estimation of drug response in clinical samples

**Fig. 9:**
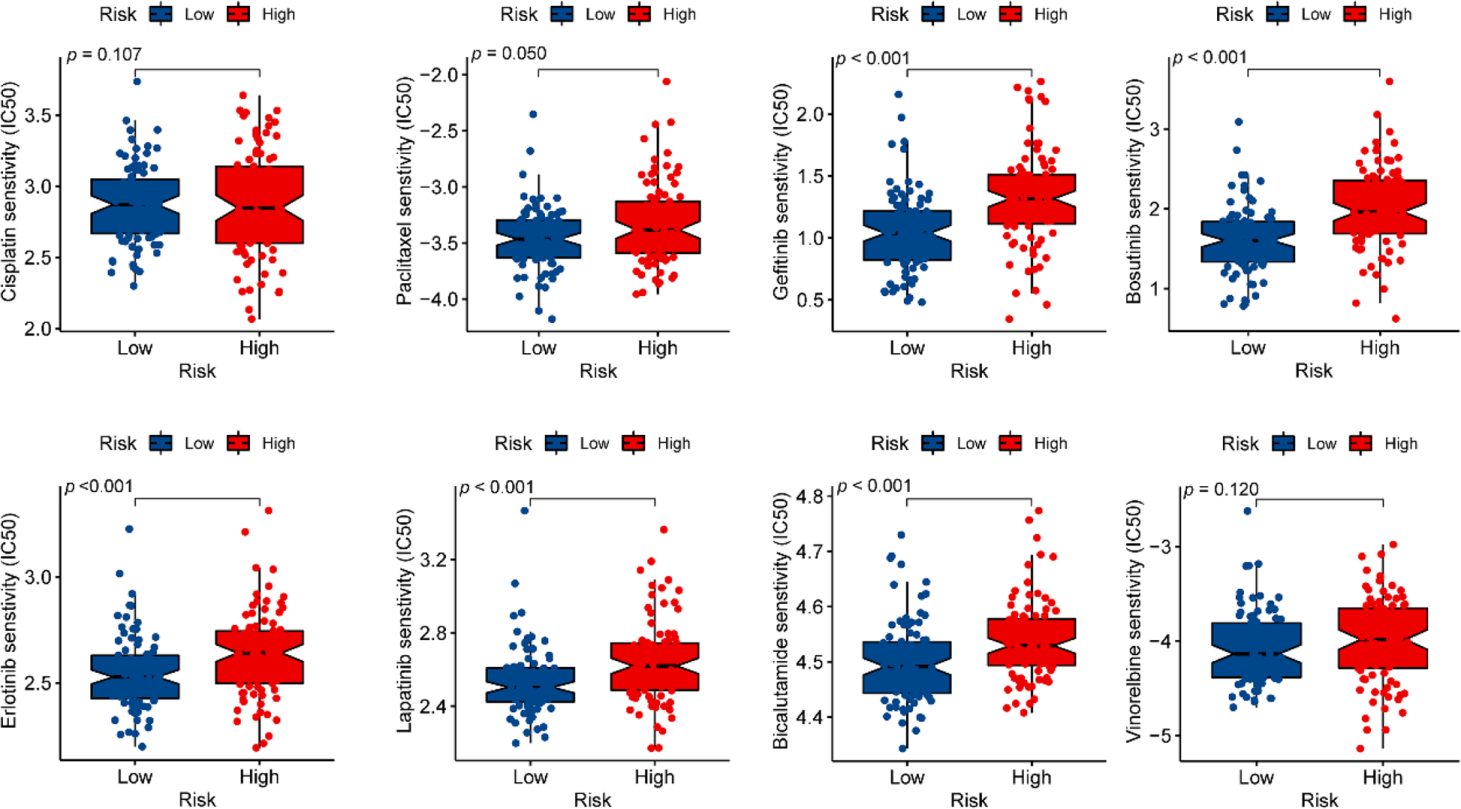
Differences in sensitivity to chemotherapeutic agents between high-risk and low-risk groups

We used IC50 to evaluate chemotherapeutic drug sensitivity, including Cisplatin, Paclitaxel, Gefitinib, Bosutinib, Erlotinib, Lapatinib, Bicalutamide, and Vinorelbine. The results demonstrated that the IC50 of Paclitaxel, Gefitinib, Bosutinib, Erlotinib, Lapatinib and Bicalutamide were significantly different in the high- and low-risk groups (Fig. 9). It can be seen that these drugs were more sensitive in the low-risk group. Thus, these drugs were not recommended for high-risk patients to chemotherapy.

## Discussion

Numerous studies have reported that chemokine modification events play an important role in tumor progression, such as, promoting cancer cell differentiation, tumor formation, and metastasis [34]. Studies have also highlighted that chemokines are involved in a variety of biological processes, including stem cell renewal, immune response, drug resistance, and tumor microenvironment remodeling [35]. Notably, tumor cells can produce many exosomes that may contain chemokines and lncRNAs, which can transmit signals between tumor cells and promote their growth and metastasis [36]. For example, Yura M et al. [37] found that the increased expression level of CCR7 in ESCC cells consequently increased their invasive ability and malignancy, which may result in a worse prognosis for ESCC patients. Guo J et al. [38] revealed that CXCL12/CXCR7 regulates EMT and other malignant processes by activating the STAT3 pathway to accelerate the growth and metastasis of esophageal cancer. A novel prognostic chemokine-related lncRNA model may improve monitoring and management of malignancies including ESCC.

In this study, we first screened chemokine-related lncRNAs by differential analysis, Pearson correlation, and LASSO regression. We then constructed a risk model for prognostic chemokine-related lncRNAs and validated the validity and accuracy of this model in predicting survival in ESCC patients. Further, our analysis discovered that the risk model played a crucial role in predicting immune cell infiltration, pathway enrichment, stromal score, and drug sensitivity in ESCC patients. In addition, we further analyzed the prognostic value and expression of each lncRNA in the ESCC patient model. This study provided clues to the progression and treatment of ESCC by comprehensively analyzing the characteristics of prognostic chemokine-related lncRNA associated with the immune environment. To further investigate the role and value of chemokine-related lncRNAs in the pathogenesis of ESCC, we used LASSO-Cox regression analysis to identify six chemokine-related lncRNAs and construct a risk model. In this study, we found that six prognostic chemokine-related lncRNAs were expressed at different levels in tumor tissues and paired adjacent normal tissues, including LINC00675, PRG1, ROR1-AS1, ANKRD10-IT1, UCA1, and EWSAT1. Interestingly, similar to our study, Zhong YB et al. [39] also found that LINC00675 expression was significantly down-regulated in both ESCC tissues and cell lines. In addition, LINC00675 may serve as an independent predictor of overall survival in ESCC. Importantly, in vitro experiments showed that LINC00675 significantly inhibited ESCC cell proliferation, colony formation, migration, invasion, and EMT, and promoted apoptosis by inhibiting the Wnt/β-catenin signaling pathway. Previous studies have also found that overexpression of UCA1 played anticancer roles in esophageal cancer cells through inhibiting cell proliferation, invasion and migration, colony formation, and cell cycle progression [40]. Further, mRNA microarray analysis of overexpressed UCA1 in EC109 cells revealed that abnormal expression of UCA1 also inhibited the Wnt signaling pathway [41].These findings indicated the potential of UCA1 as a biomarker and its effect on suppressing the pathogenesis and progression of esophageal cancer in vitro and in vivo. Wang X et al. [42] demonstrated that N-nitrosamines (NAs)-mediated downregulation of UCA1 promoted ESCC progression through targeting hnRNP F/FGFR2/PI3k-AKT axis, which provides a new chemical carcinogenic target. The above findings well explain that some lncRNAs associated with chemokines are overexpressed in tumors and act as oncogenes, while others are highly expressed as tumor suppressor genes in paired adjacent normal tissues. Also, we found that six lncRNAs were expressed differently in the high-risk and low-risk groups. These findings further suggest that lncRNAs in risk model have favorable research value.

Indeed, recent studies have reported that chemokine modifications and multiple lncRNAs can participate in multiple processes of tumor development [43, 44]. Therefore, in order to explore whether risk models play a role in tumor and TME, we first performed GSEA and GO/KEGG enrichment analysis. We demonstrated that multiple cancer- and immune-related pathways were associated with the risk model, including calcium signaling, cell adhesion, DNA replication, melanogenesis, type II diabetes, WNT signaling, herpes simplex virus 1 infection, vascular smooth muscle contraction, cytokine-cytokine receptor interaction, signaling pathways regulating pluripotency of stem cell. In the future, further biological experiment is required to clarify these bioinformatics analyses. Yet these classical signaling regulation pathways still imply the complexity of the ESCC TME ecosystem. Also, previous studies have found that cancer-related pathways could regulate the ESCC development [37, 40]. For example, Fujikawa M et al. [45] indicated that the interaction between stromal CCL1 and CCR8 on cancer cells promotes ESCC progression via the Akt/proline-rich Akt substrate of 40 kDa/mammalian target of rapamycin pathway.

The immune system plays important role in the development of cancers, as well as immunotherapy [46]. On this basis, we suggested that the risk model may influence tumor immune processes in ESCC, including processes including penetration of immune cells. Our findings that high levels of infiltration of macrophages M2 and monocytes were positively correlated in high-risk group, which suggested that these cells are more permeable in high-risk patients. Previous studies have also found that M2 macrophages and monocytes play a tumor-promoting role. On the one hand, monocytes can influence the tumor microenvironment through various mechanisms, thereby inducing angiogenesis, immune tolerance, and dissemination of tumor cells [47]. On the other hand, the massive penetration of macrophages M2 into solid tumors are related to EMT, tumor progression and distant metastasis, resulting in low patient survival and poor treatment outcomes [48]. Afterward, we revealed that ESCC patients in high-risk group had higher immune scores, stromal scores, ESTIMATE scores, and lower tumor purity than those in low-risk group, suggesting a higher degree of immune infiltration in high-risk group than in low-risk group. These findings are similar to previous studies in that they also found that patients with tumors with high immune and stromal scores had lower overall survival, i.e., poorer prognosis [49, 50]. It has also been found in the literature that more tumor-infiltrating immune cells in high-risk group were associated with an increased risk of recurrence and worse survival [51]. Therefore, we hypothesized that unresponsiveness and higher immunosuppression in the TME will lead to worse survival in high-risk patients. These results supported this risk model as a predictor of immune status in ESCC patients.

In addition, risk scores were also significantly associated with sensitivity to multiple targeted agents, including the commonly used chemotherapeutic agents, such as, Paclitaxel, Gefitinib, Bosutinib, Erlotinib, Lapatinib, Bicalutamide, and some new drugs in clinical practice. These data suggested that this predictive chemokine-related lncRNAs risk model had potential practical value in assessing the efficacy and sensitivity of various drugs.

Despite our positive findings, we recognized that there were still some limitations in our study. Firstly, the sample size of this study was limited, and in the next step, we will expand the sample size and do in-depth study of chemokine-related lncRNA signature for ESCC patients. Secondly, external validation with large sample size was not performed, which might cause a risk of overfitting. In this regard, we conducted 500 times LASSO regression analysis to adjust the parameters. Thirdly, the potential molecular mechanisms and biological functions of this prognostic model remained uncertain, and experimental studies were needed to verify these findings.

## Conclusions

In conclusion, we developed a novel risk model of prognostic chemokine-related lncRNAs and then validated the validity and accuracy of it in predicting survival for ESCC patients. This model also elucidated the crucial role of novel prognostic chemokine-related lncRNAs in prognosis, immune landscape, and drug therapy, thereby providing insights for prognosis prediction and personalized treatment strategies in ESCC.

## Supplementary Information

**Additional file 1:** **Table S1.** List of 64 chemokines. **Figure S1.** The expression of six lncRNA in the high-risk group and low-risk group (*p<0.05, **p<0.01, ***p<0.001). **Figure S2**. Subgroup Analyses of KM Analysis stratified by age, sex and stage. **Figure S3.** ROC of in risk model in the testing set. ROC, receiver operating characteristic curve; AUC, area under the curve. **Figure S4.** DCA curve of the risk model in the testing set. DCA, decision curve analysis. **Figure S5.** Calibration curve of the risk model in the training set. OS, overall survival. **Figure S6.** ROC of the prognostic model in the training set. ROC, receiver operating characteristic curve; AUC, area under the curve. **Figure S7.** DCA curve of the prognostic model in the training set. **Figure S8.** Calibration curve of the prognostic model in the training set. **Figure S9.** ROC of the prognostic model in the testing set. ROC, receiver operating characteristic curve; AUC, area under the curve. **Figure S10.** DCA curve of the prognostic model in the testing set. **Figure S11.** Calibration curve of the prognostic model in the testing set. **Figure S12.** GO/KEGG enrichment analysis.

## Data Availability

All RNA-seq data was obtained from NCBI GEO dataset with the accession number (GSE53624 and GSE53622). “The datasets generated and/or analysed during the current study are available in the NCBI_GEO repository https://www.ncbi.nlm.nih.gov/geo/query/acc.cgi?acc=GSE53624. The immune cell infiltration was calculated by the R package entitled “CIBERSORT” analysis. https://www.ncbi.nlm.nih.gov/geo/query/acc.cgi?acc=GSE53624; https://www.ncbi.nlm.nih.gov/geo/query/acc.cgi.
