# Supplementary material for: Novel chemokine related LncRNA signature correlates with the prognosis, immune landscape, and therapeutic sensitivity of esophageal squamous cell cancer
Source: BMC Gastroenterol. 2023 Apr 20;23:132. doi: 10.1186/s12876-023-02688-5 (PMC10120245; doi:10.1186/s12876-023-02688-5)
Supplement: Supplementary file 1 — Additional file 1: Table S1. List of 64 chemokines. Figure S1. The expression of six lncRNA in the high-risk group and low-risk group (*p<0.05, **p<0.01, ***p<0.001). Figure S2. Subgroup Analyses of KM Analysis stratified by age, sex and stage. Figure S3. ROC of in risk model in the testing set. ROC, receiver operating characteristic curve; AUC, area under the curve. Figure S4. DCA curve of the risk model in the testing set. DCA, decision curve analysis. Figure S5. Calibration curve of the risk model in the training set. OS, overall survival. Figure S6. ROC of the prognostic model in the training set. ROC, receiver operating characteristic curve; AUC, area under the curve. Figure S7. DCA curve of the prognostic model in the training set. Figure S8. Calibration curve of the prognostic model in the training set. Figure S9. ROC of the prognostic model in the testing set. ROC, receiver operating characteristic curve; AUC, area under the curve. Figure S10. DCA curve of the prognostic model in the testing set. Figure S11. Calibration curve of the prognostic model in the testing set. Figure S12. GO/KEGG enrichment analysis. [file 12876_2023_2688_MOESM1_ESM.docx]

| Table S1 List of 64 chemokines | | |
| --- | --- | --- |
| Names | Tumor promoting/inhibitory effect | Key immune function of chemokine receptors |
| CCL1 | Tumor promoting effect |  |
| CCL11 | Tumor promoting effect |  |
| CCL13 | Tumor promoting effect |  |
| CCL14 | Tumor promoting effect |  |
| CCL15 | Tumor promoting effect |  |
| CCL16 | Tumor inhibitory effect |  |
| CCL17 | Tumor promoting/inhibitory effect |  |
| CCL18 | Tumor promoting effect |  |
| CCL19 | Tumor inhibitory effect |  |
| CCL2 | Tumor promoting effect |  |
| CCL20 | Tumor promoting effect |  |
| CCL21 | Tumor inhibitory effect |  |
| CCL22 | Tumor promoting effect |  |
| CCL23 | Tumor inhibitory effect |  |
| CCL24 | Tumor promoting effect |  |
| CCL25 | Tumor promoting effect |  |
| CCL26 | Tumor promoting effect |  |
| CCL27 | Tumor promoting/inhibitory effect |  |
| CCL28 | Tumor promoting effect |  |
| CCL3 | Tumor promoting effect |  |
| CCL3L1 | Tumor promoting effect |  |
| CCL4 | Tumor promoting effect |  |
| CCL4L2 | Tumor promoting effect |  |
| CCL5 | Tumor promoting effect |  |
| CCL7 | Tumor promoting effect |  |
| CCL8 | Tumor promoting/inhibitory effect |  |
| CCR1 | Tumor promoting effect | Innate immunity Adaptive immunity |
| CCR2 | Tumor promoting effect | Monocyte trafficking Th1-type adaptive immunity |
| CCR3 | Tumor promoting effect | Th2-type adaptive immunity Eosinophil distribution and trafficking |
| CCR4 | Tumor promoting effect | Homing of T cells to skin and lung Th2-type immune response |
| CCR5 | Tumor promoting effect | Type-1 adaptive immunity |
| CCR6 | Tumor promoting effect | iDC trafficking, GALT development Th17 adaptive immune responses |
| CCR7 | Tumor promoting effect | mDC, and B- and T-cell trafficking in lymphoid tissue to T-cell zone, Egress of DC and T cells from tissue |
| CCR8 | Tumor promoting effect | Immune surveillance in skin type-2 adaptive immunity, thymopoiesis |
| CCR9 | Tumor promoting effect | Homing of T cells to gut GALT development and function, thymopoiesis |
| CCR10 | Tumor promoting effect | Humoral immunity at mucosal sites Immune surveillance in skin |
| CCRL2 | Tumor promoting effect |  |
| CX3CL1 | Tumor promoting/inhibitory effect |  |
| CX3CR1 | Tumor inhibitory effect |  |
| CXCL1 | Tumor promoting effect |  |
| CXCL10 | Tumor promoting/inhibitory effect |  |
| CXCL11 | Tumor promoting effect |  |
| CXCL12 | Tumor promoting effect |  |
| CXCL13 | Tumor promoting effect |  |
| CXCL14 | Tumor promoting/inhibitory effect |  |
| CXCL16 | Tumor promoting effect |  |
| CXCL17 | Tumor promoting effect |  |
| CXCL2 | Tumor promoting effect |  |
| CXCL3 | Tumor promoting effect |  |
| CXCL5 | Tumor promoting effect |  |
| CXCL6 | Tumor promoting effect |  |
| CXCL8 | Tumor promoting/inhibitory effect |  |
| CXCL9 | Tumor promoting/inhibitory effect |  |
| CXCR1 | Tumor promoting effect | Neutrophil trafficking |
| CXCR2 | Tumor inhibitory effect | B-cell lymphopoiesis, Neutrophil egress from bone marrow Neutrophil trafficking |
| CXCR3 | Tumor inhibitory effect | Th1-type adaptive immunity |
| CXCR4 | Tumor promoting effect | Hematopoiesis Organogenesis Bone marrow homing |
| CXCR5 | Tumor promoting effect | B- and T-cell trafficking in lymphoid tissue to B-cell zone/follicles |
| CXCR6 | Tumor inhibitory effect | Innate lymphoid cell function Adaptive immunity |
| PF4 | Tumor inhibitory effect |  |
| PF4V1 | Tumor inhibitory effect |  |
| XCL1 | Tumor inhibitory effect |  |
| XCL2 | Tumor promoting effect |  |
| XCR1 | Tumor promoting effect |  |


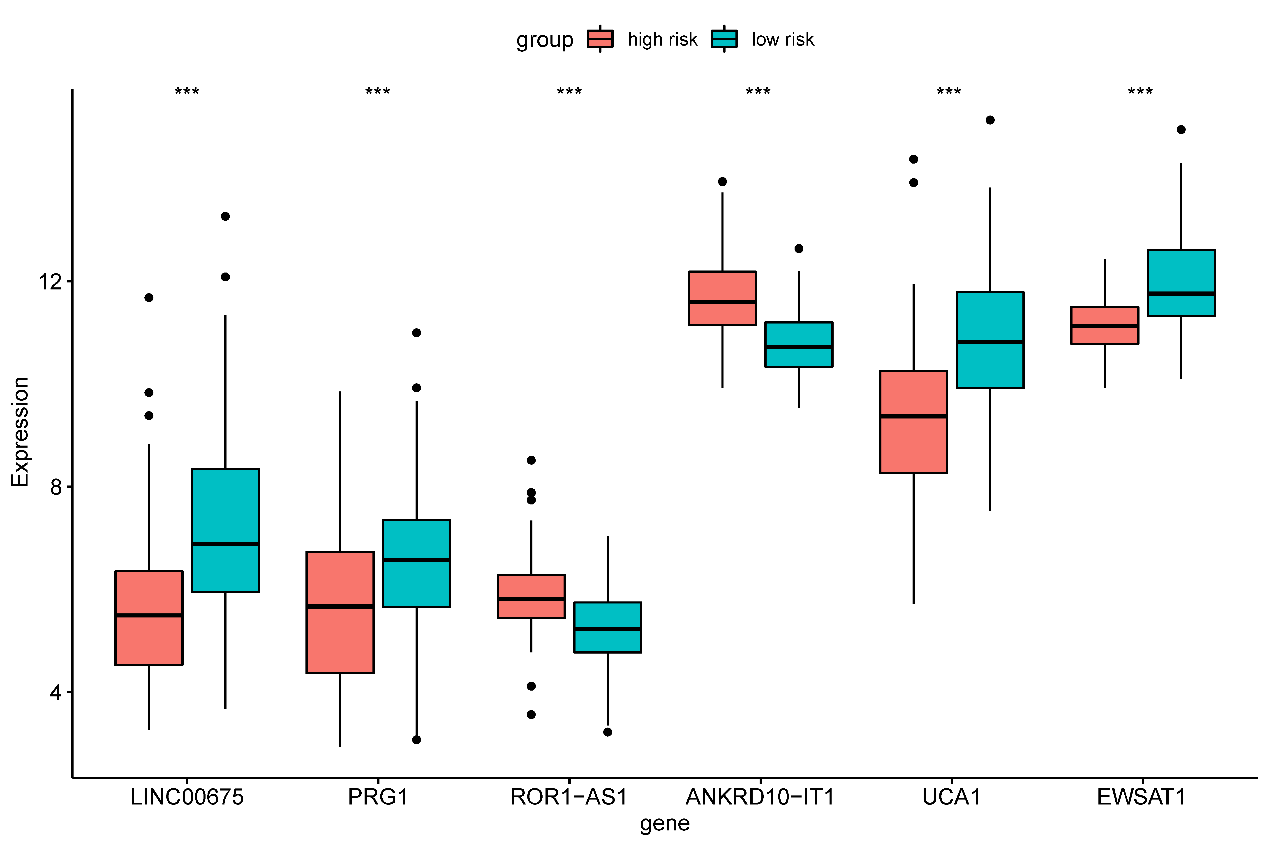


Figure S1 The expression of six LncRNA in the high-risk group and low-risk group (*p<0.05, **p<0.01, ***p<0.001)


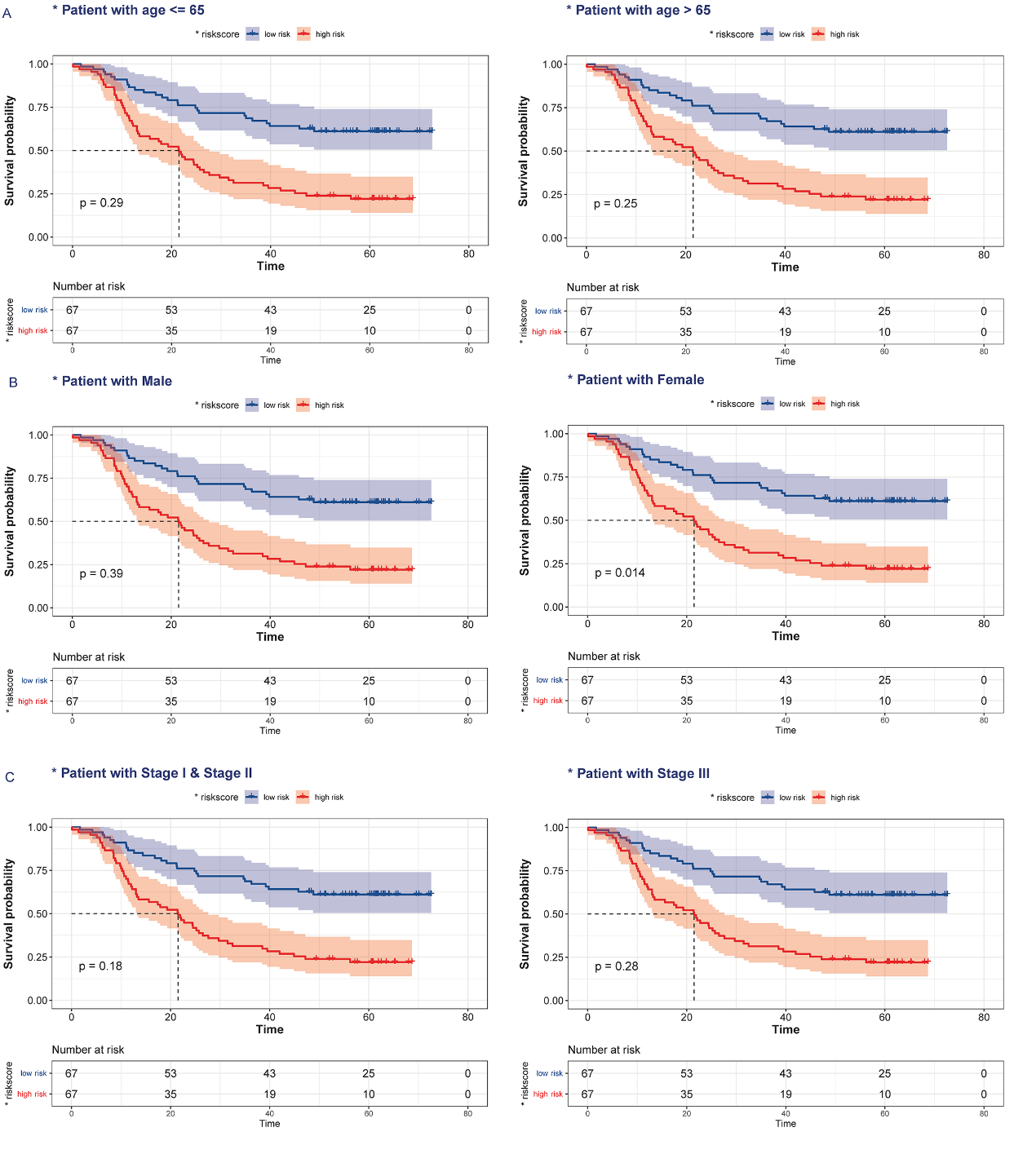


Figure S2 Subgroup Analyses of KM Analysis stratified by age, sex and stage.


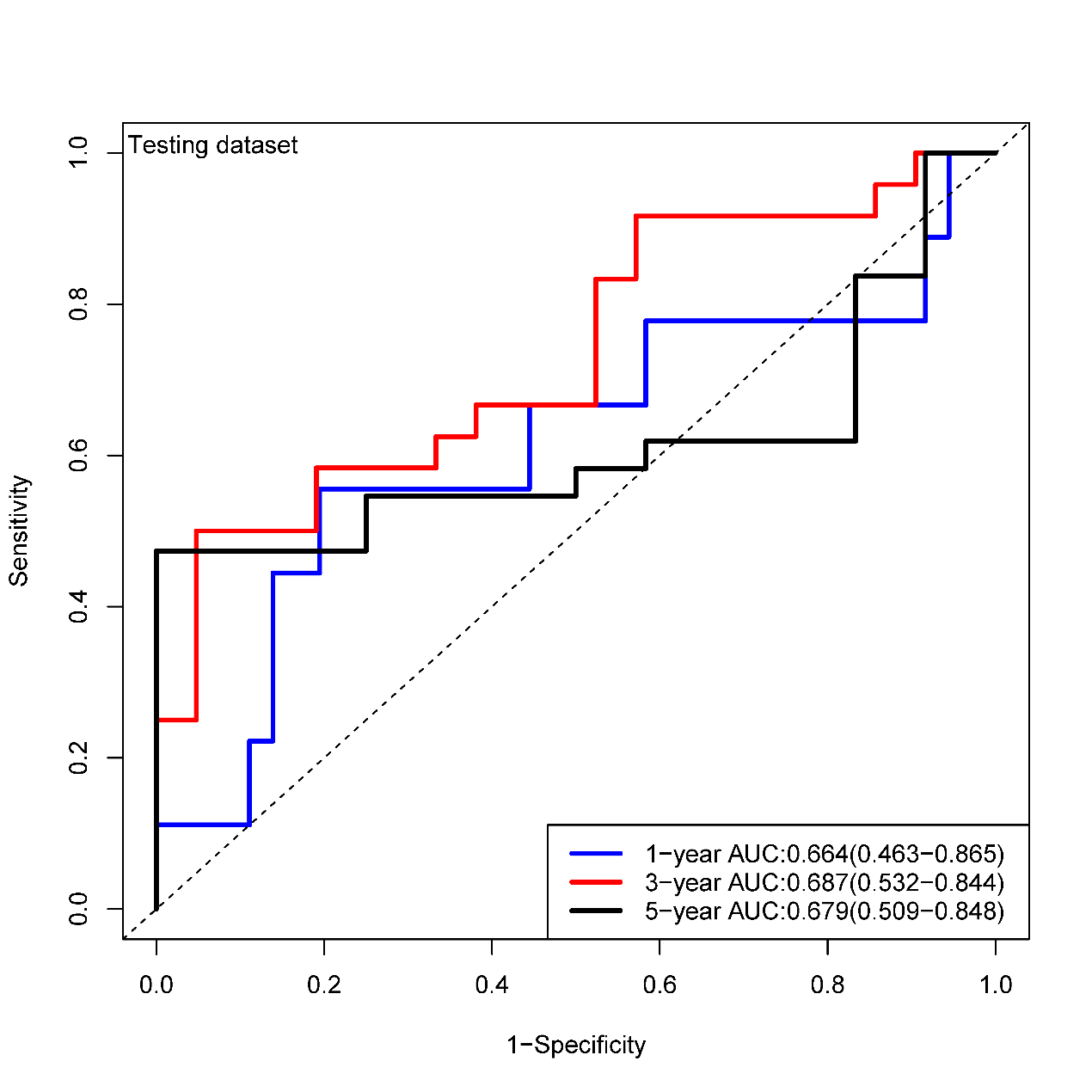


Figure S3 ROC of in risk model in the testing set.

ROC, receiver operating characteristic curve; AUC, area under the curve


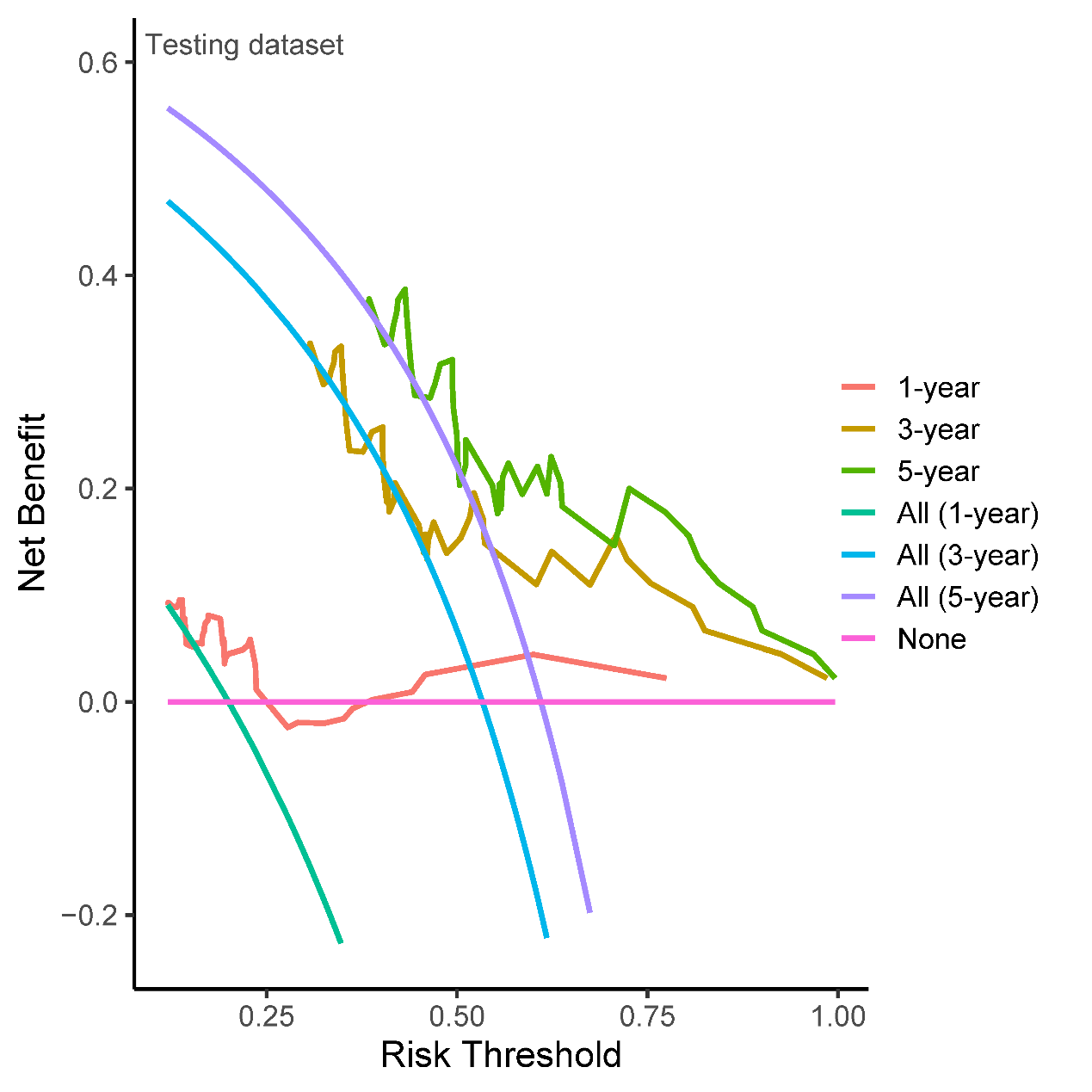


Figure S4 DCA curve of the risk model in the testing set.

DCA, decision curve analysis


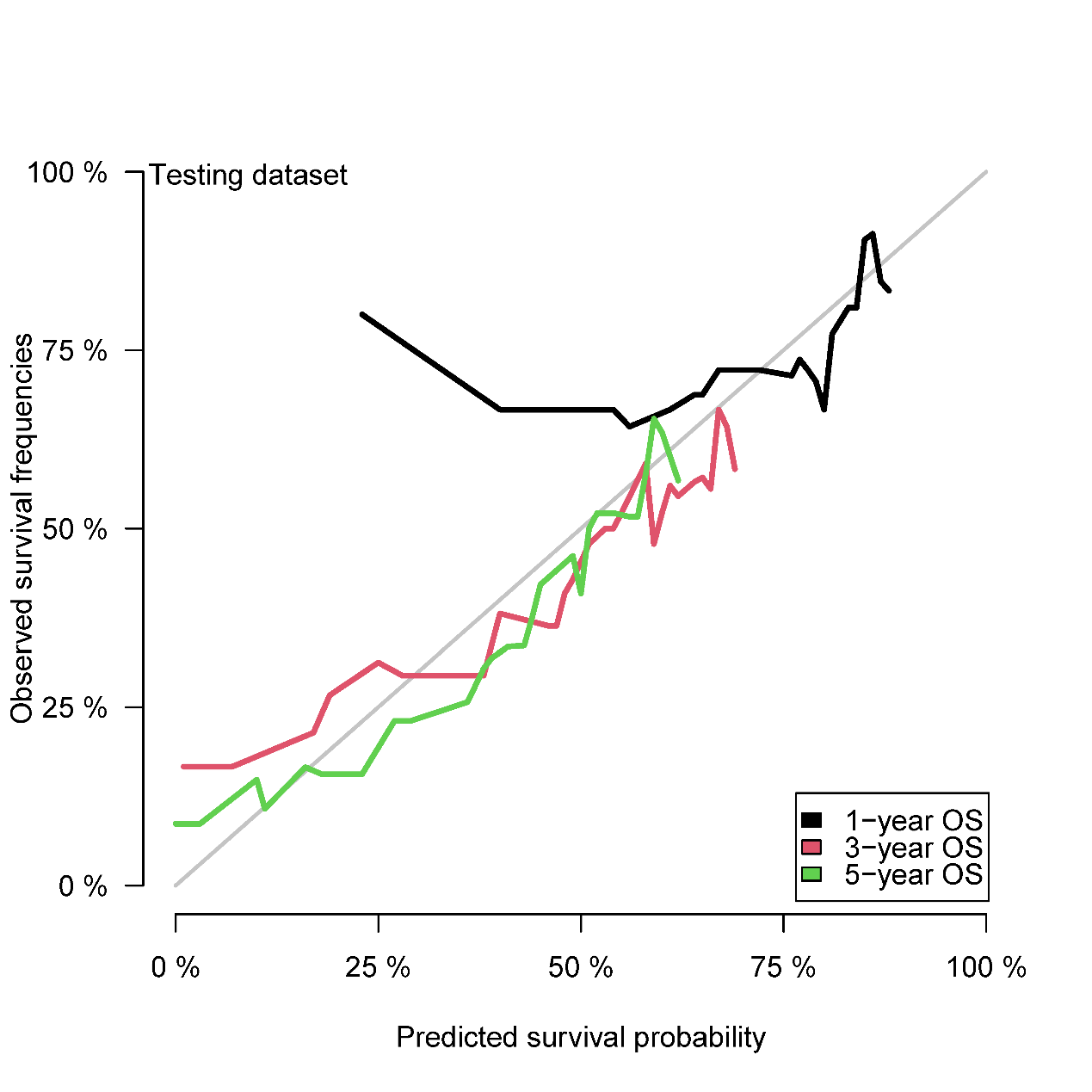


Figure S5 Calibration curve of the risk model in the training set.

OS, overall survival


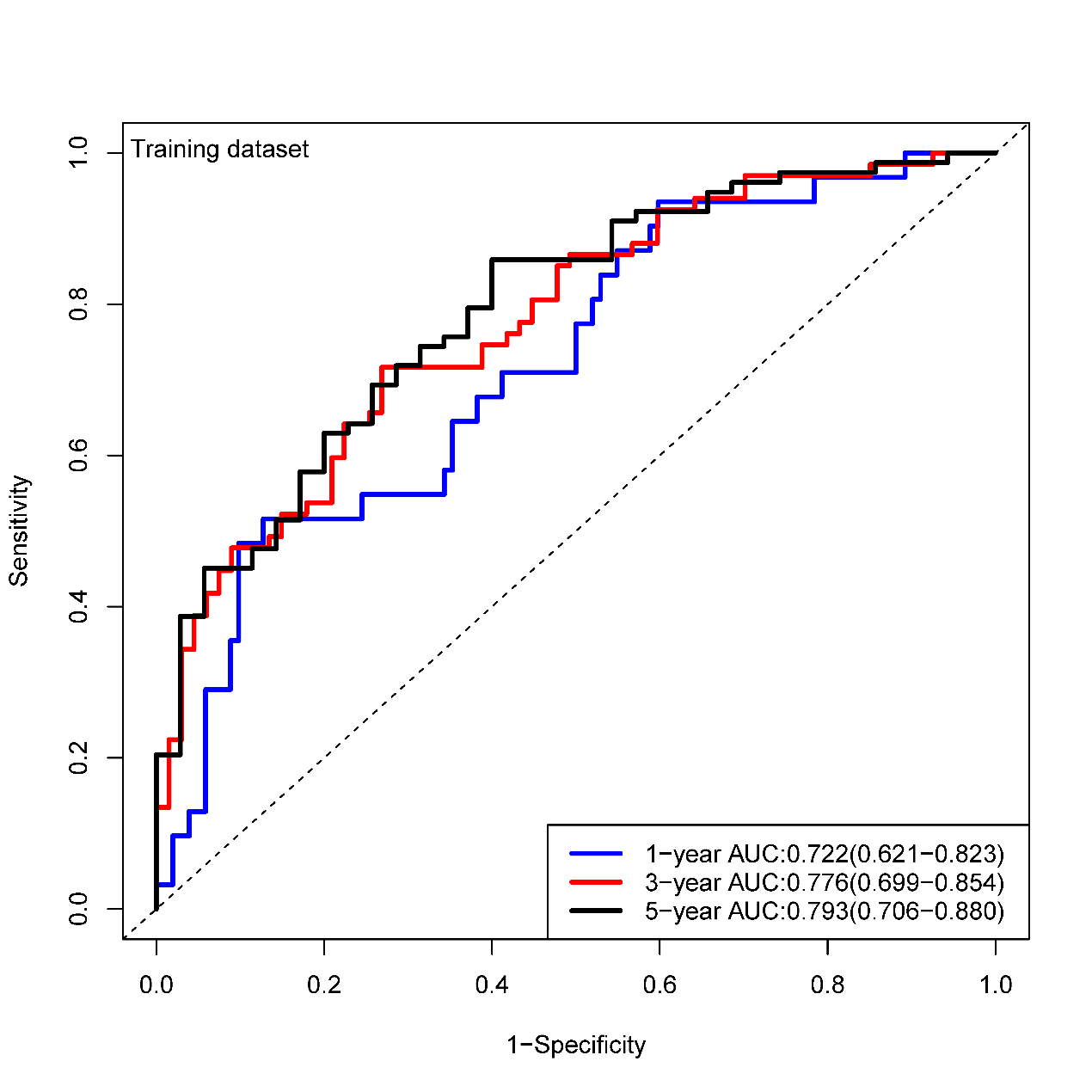


Figure S6 ROC of the prognostic model in the training set.

ROC, receiver operating characteristic curve; AUC, area under the curve


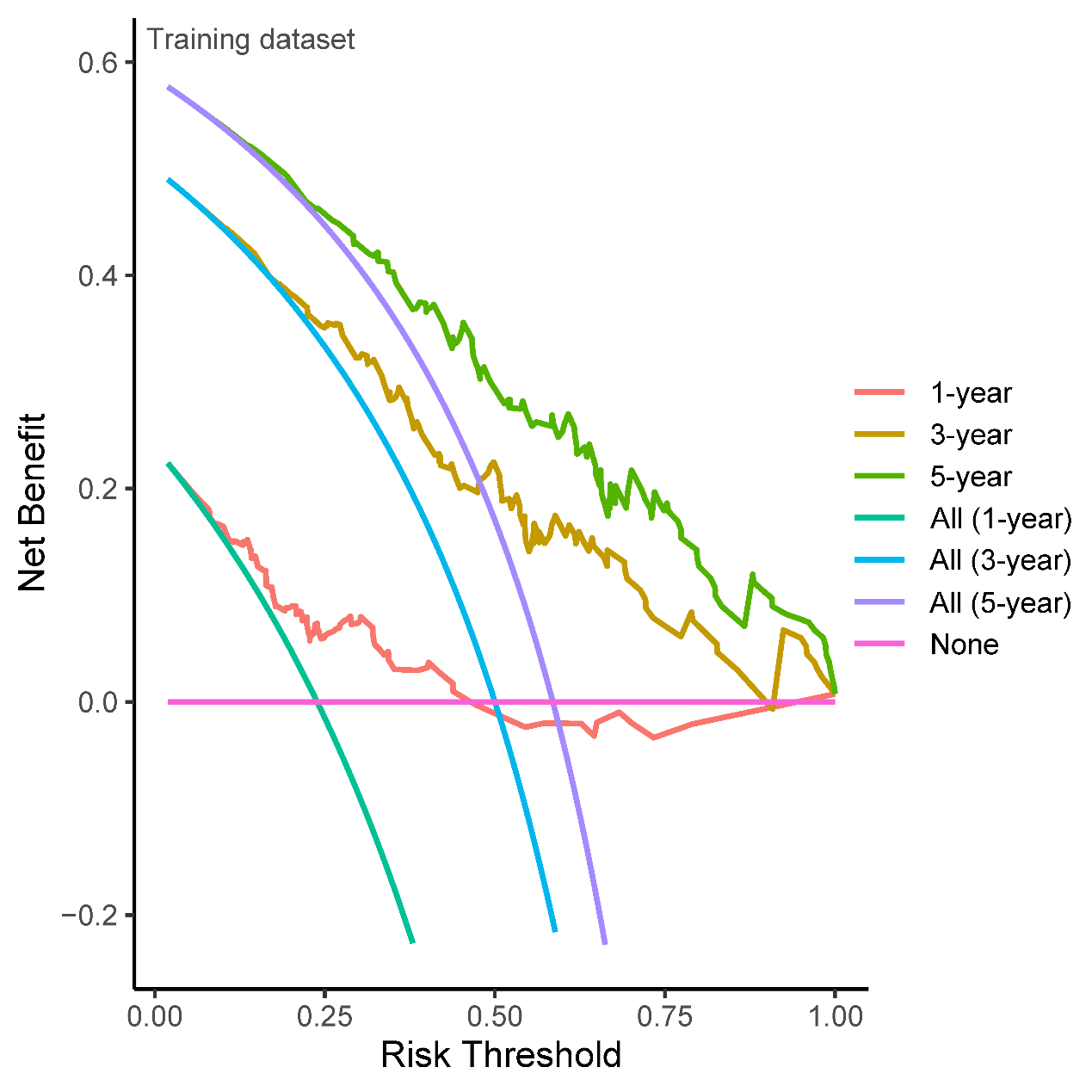


Figure S7 DCA curve of the prognostic model in the training set.


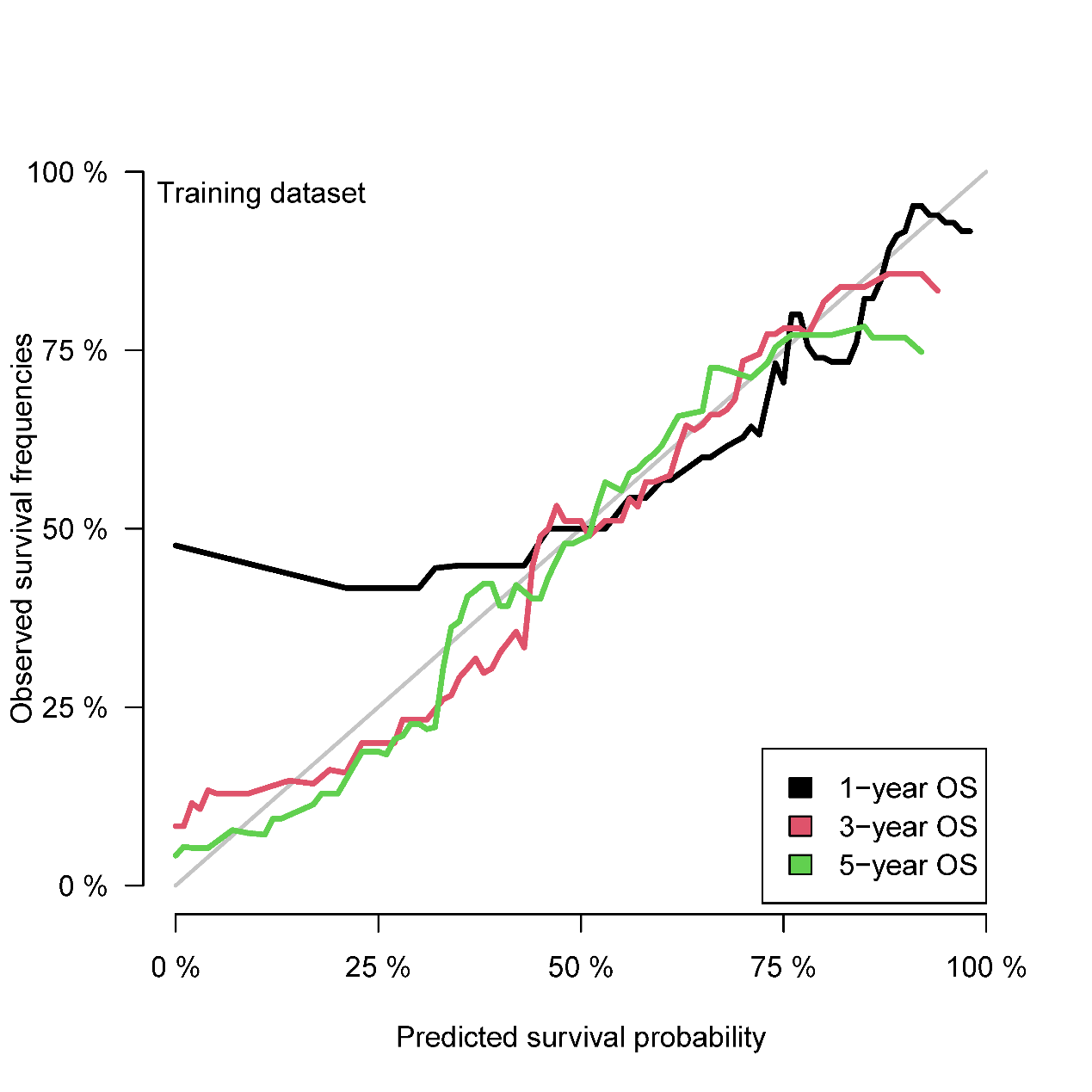


Figure S8 Calibration curve of the prognostic model in the training set.


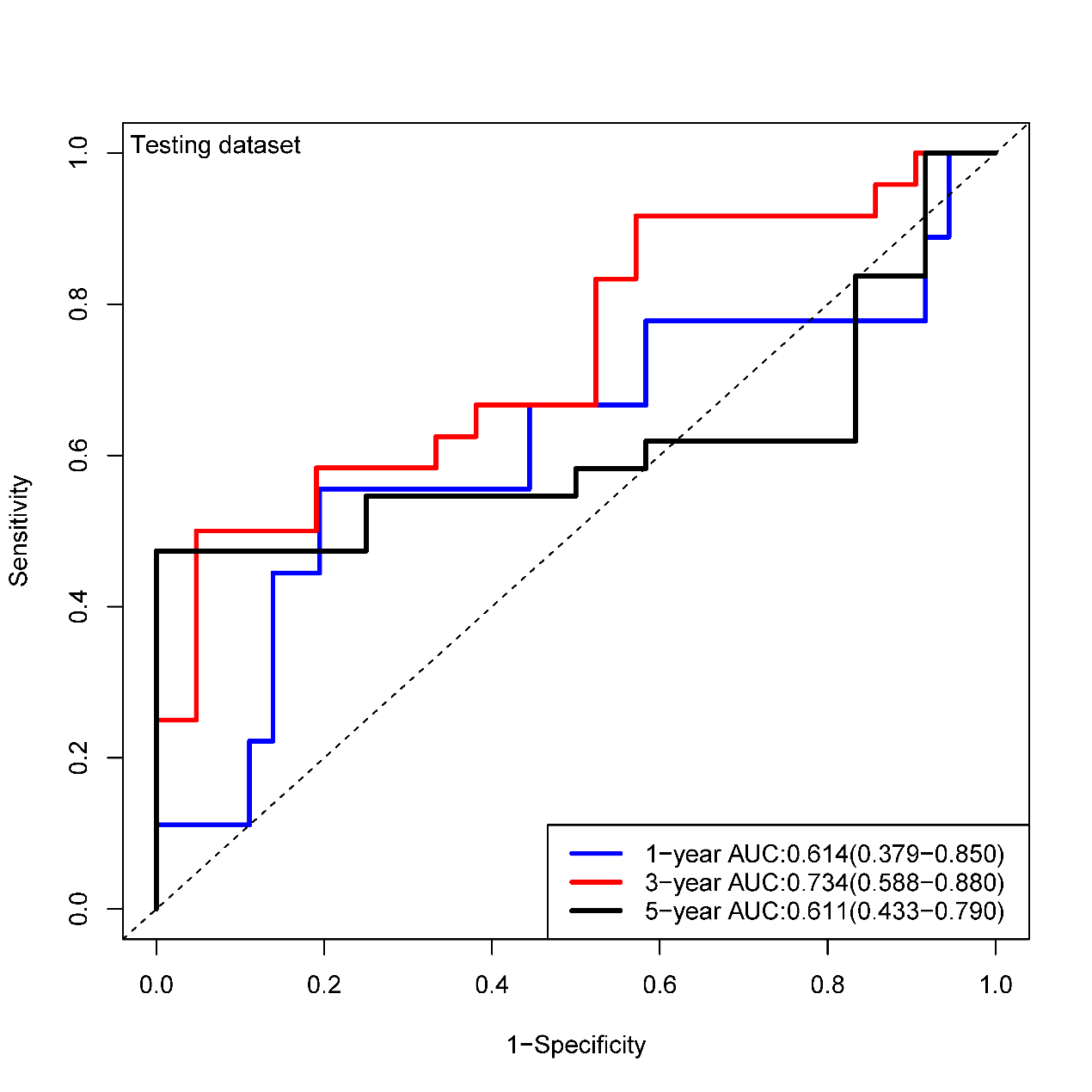


Figure S9 ROC of the prognostic model in the testing set.

ROC, receiver operating characteristic curve; AUC, area under the curve


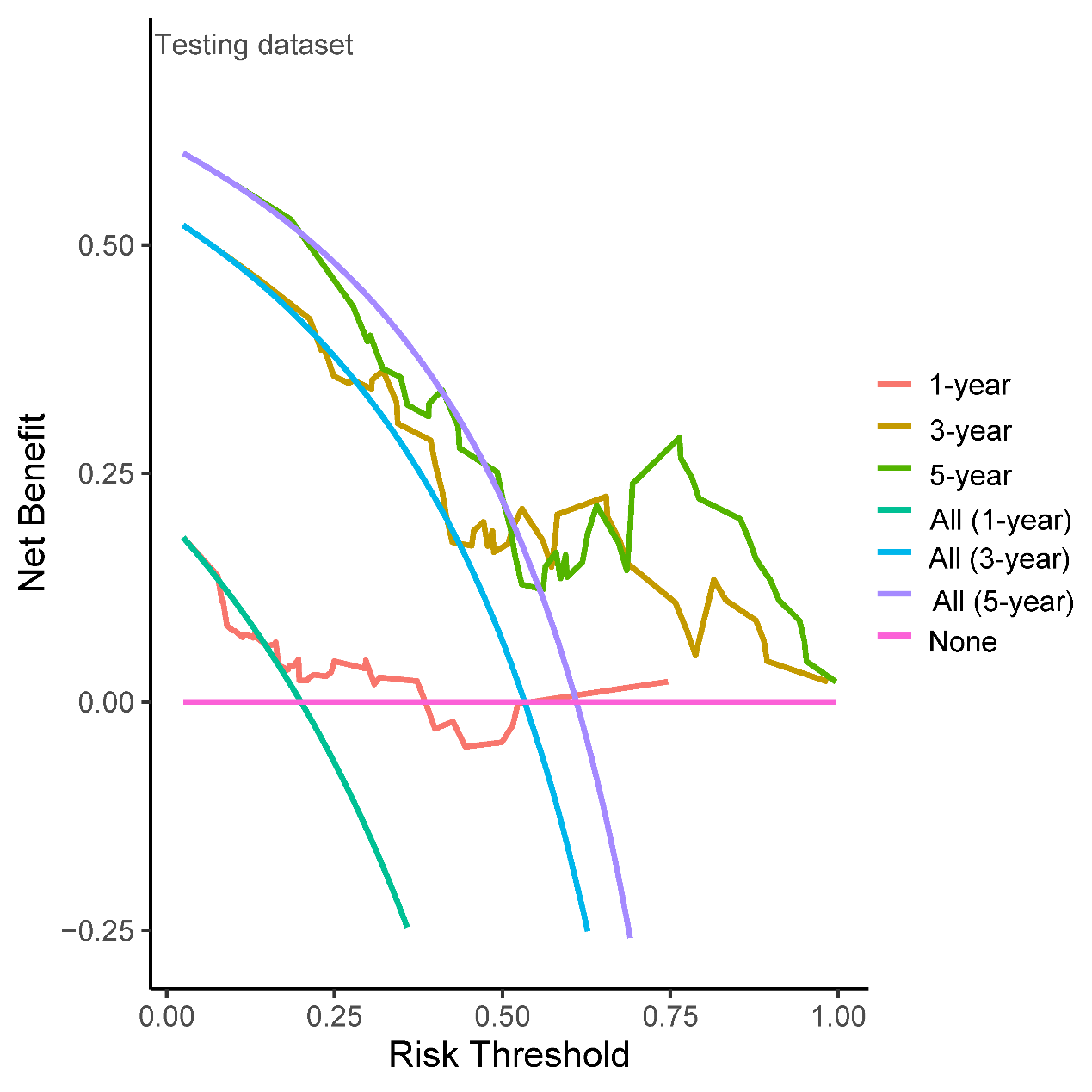


Figure S10 DCA curve of the prognostic model in the testing set.


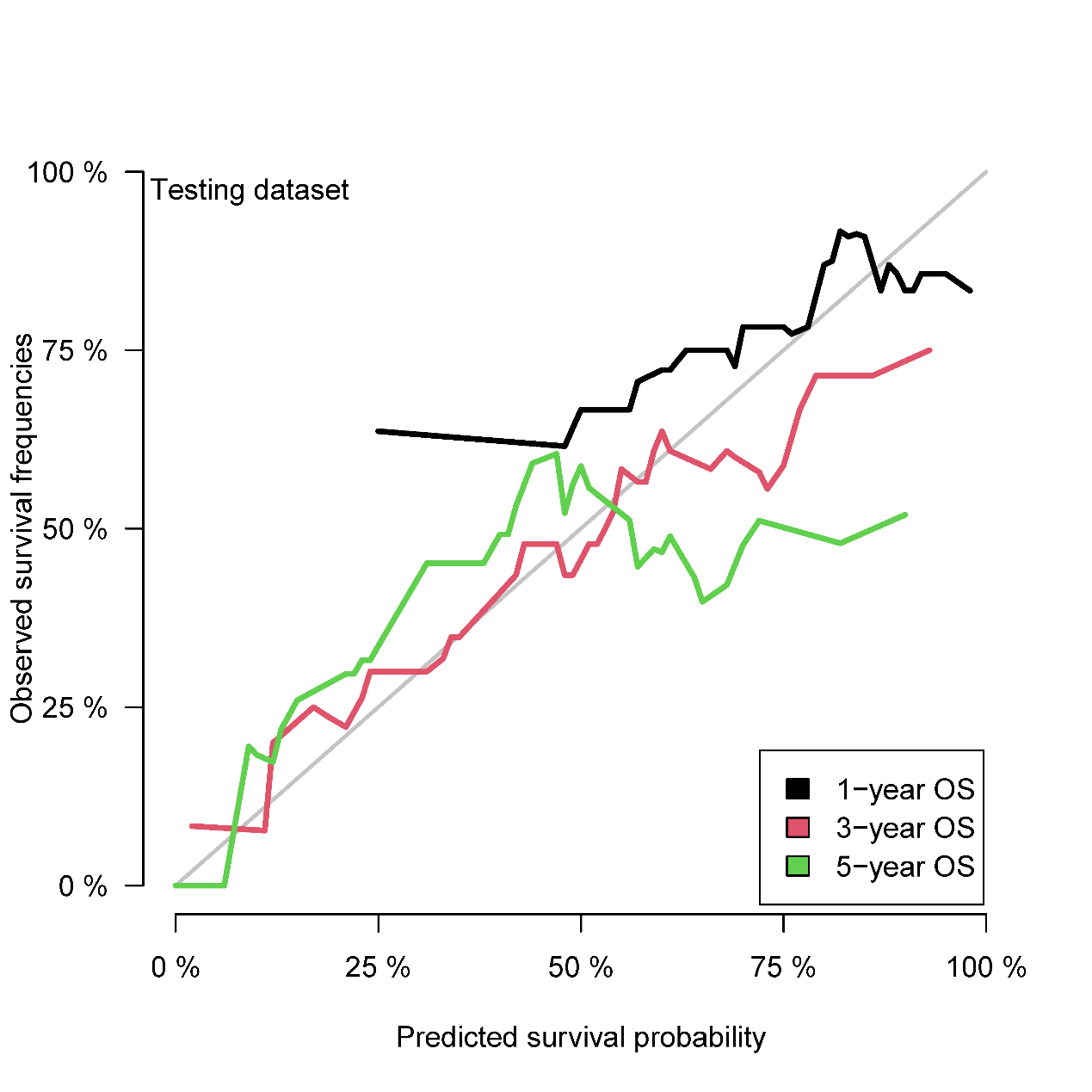


Figure S11 Calibration curve of the prognostic model in the testing set.


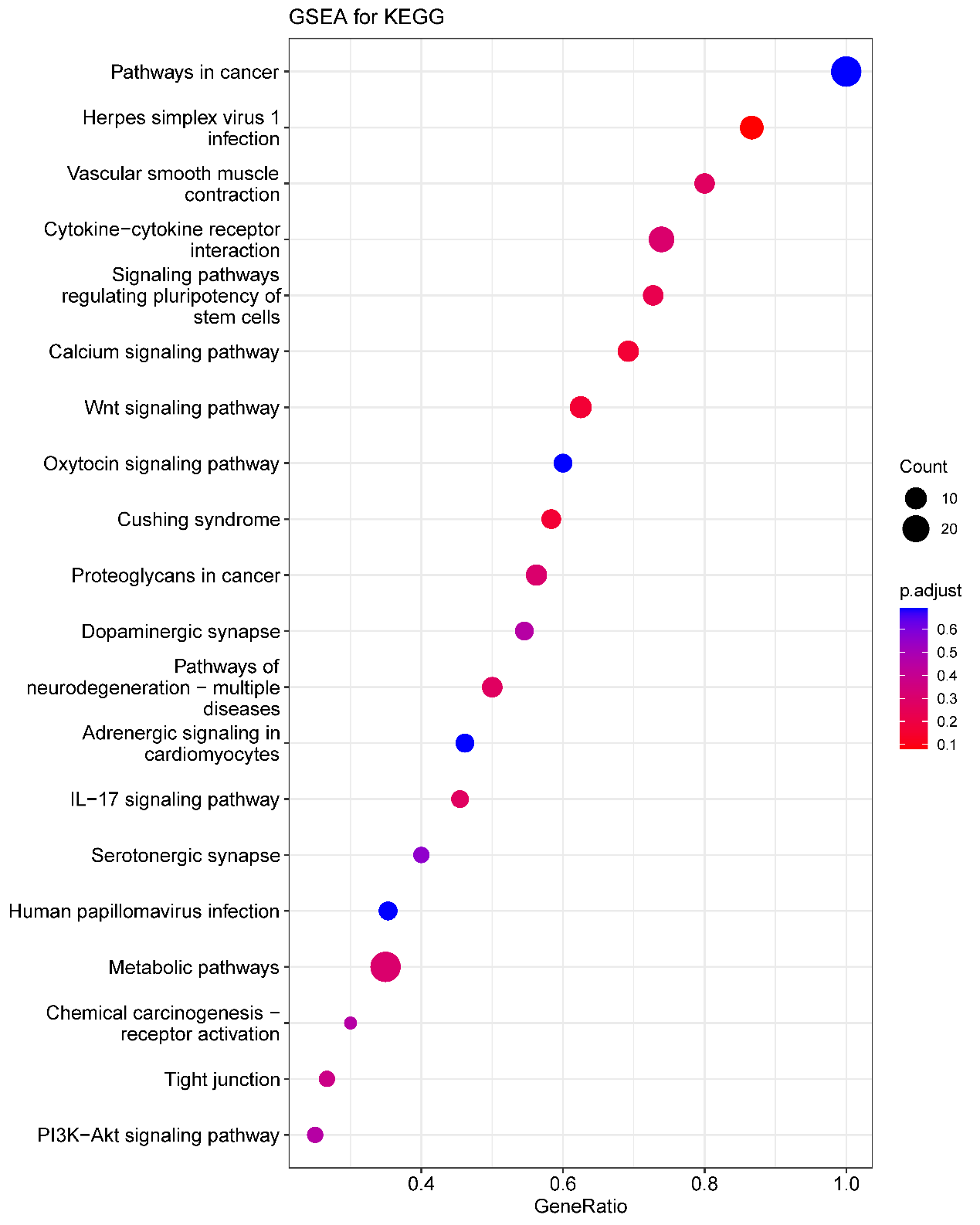


Figure S12 GO/KEGG enrichment analysis.
